# Influence of different feeding regimes on the survival, growth, and biochemical composition of *Acropora* coral recruits

**DOI:** 10.1371/journal.pone.0188568

**Published:** 2017-11-28

**Authors:** Jessica A. Conlan, Craig A. Humphrey, Andrea Severati, David S. Francis

**Affiliations:** 1 Deakin University, Geelong, Australia, School of Life and Environmental Sciences, Warrnambool Campus, Warrnambool, Victoria, Australia; 2 The National Sea Simulator, Australian Institute of Marine Science, Townsville, Queensland, Australia; Leibniz Centre for Tropical Marine Research, GERMANY

## Abstract

Heterotrophic feeding in newly-settled coral planulae can potentially improve survivorship and accelerate early development in some species; however, an optimal diet to facilitate this does not currently exist. This study evaluated the efficacy of three heterotrophic feeding regimes (enriched rotifers, unfiltered seawater, and a novel, particulate diet), against a wholly-phototrophic treatment on *Acropora hyacinthus*, *A*. *loripes*, *A*. *millepora*, and *A*. *tenuis* recruits, over 93 days post-settlement. The unfiltered seawater treatment recorded maximum survival for all species (*A*. *hyacinthus* 95.9±8.0%, *A*. *loripes*: 74.3±11.5%, *A*. *millepora*: 67±12.7%, *A*. *tenuis*: 53.2±11.3%), although not significant. Growth (% surface area gain) was also greatest in the unfiltered seawater, and this was significant for *A*. *millepora* (870±307%) and *A*. *tenuis* (693±91.8%) (*p*<0.05). Although total lipid concentration was relatively stable across treatments, the lipid class composition exhibited species-specific responses to each treatment. Lower saturated and higher polyunsaturated fatty acids appeared beneficial to recruit performance, particularly in the unfiltered seawater, which generally contained the highest levels of 20:5n-3 (EPA), 22:6n-3 (DHA), and 20:4n-6 (ARA). The present study demonstrates the capacity of a nutritionally adequate and readily accepted heterotrophic feeding regime to increase coral recruit survival, growth, and health, which can greatly reduce the time required in cost- and labour-intensive culture.

## Introduction

As global coral reef degradation intensifies as a consequence of anthropogenic activities, coral aquaculture has gained recognition for its potential to mass-produce coral propagules for reef-rehabilitation, experimentation and the aquaria trade [1,2]. Recent advancements in coral culturing techniques have explored the use of sexually propagated larvae as source material for the mass production of scleractinian (reef-building) corals [3,4]. In particular, corals of the genus *Acropora* represent a promising candidate for aquaculture, being among the most dominant scleractinian corals in the Indo-Pacific reefs, with a high fecundity, and fast growth rates compared to other genera [5].

However, all scleractinian larvae and recruits face several challenges during early life [3]. In the wild, mortality can exceed 99% in the first year of life, largely due to competition, sedimentation, and predation [6]. *Ex situ* grow-out facilities therefore have the potential to drastically improve post-settlement survivorship by eliminating natural stressors inside a controlled environment of optimal temperature, photoperiod, water quality, and flow rate [1]. However, coral recruit aquaculture is still largely at an experimental stage, with significant mortality and poor growth in the early post-settlement period presenting a major hurdle with regard to labour- and cost-efficiency [7].

Recruit survivorship increases substantially with size, as larger corals possess a larger energy reservoir to invest into growth, immunity, and fitness, while also reducing vulnerability to whole colony mortality via resource sharing between polyps [8]. However, in order to accelerate coral recruits to a juvenile size-refuge, adequate provision of energy and requisite nutrients is essential [9]. Therefore, improved understanding of the nutritional requirements scleractinian coral early life stages is necessary to advance their culture.

A coral’s overall health and fitness can be measured via its energetic status, which refers to the energy available compared to the energetic requirements [10]. A common indicator used to assess the energetic status of corals is lipids [11,12]. Lipids provide a metabolic energy source and specific nutrients (e.g. fatty acids (FA), sterols, and phospholipids), and are typically associated with growth, metabolic fuel, membrane structure, reproduction, and immune function [13,14]. Although energy provided by the photosynthetic symbionts (zooxanthellae) can account for a large portion of lipids acquired by the coral host, many coral species possess varying degrees of ability to supplement their diet with heterotrophically derived lipids and FA [15]. Heterotrophic feeding also provides inorganic nutrients such as nitrogen and phosphorus, as well as essential amino acids, and trace vitamins and minerals that are essential for proper metabolic functioning that would otherwise not be provided by phototrophy alone [15,16]. Heterotrophic feeding enhances tissue growth, skeletal calcification rates, and lipid storage in corals [14,17]—key determinants of post-settlement success. As such, capitalisation on coral heterotrophy in *ex situ* culture could significantly improve survival rates and accelerate post-settlement development toward a juvenile size-refuge, advancing coral aquaculture prospects [2].

While there are few studies examining heterotrophy during the most critical early life stages in captive corals, some foundational investigations have recorded improved recruit growth rates following heterotrophic inputs [2,18]. However, most studies reporting the effects of coral heterotrophy *ex situ* have only considered the addition of indiscriminate exogenous feeds such as *Artemia* nauplii [12,18–20], with little consideration for the nutritional requirements of the corals, and the subsequent impact on their nutritional profiles. In particular, *Artemia* nauplii are known to be deficient in polyunsaturated fatty acids (PUFA), particularly the long-chain PUFA (LC-PUFA); 22:6n-3 (DHA) [21], which is known to be crucial for growth, survival, and early development in marine larvae [22]. Moreover, previous reports have shown that scleractinian coral eggs are deficient in DHA [23], indicating that it must be rapidly acquired following benthic settlement. Although *Artemia* can be nutritionally enriched to improve the FA profile [21], this necessitates extended culture time, resulting in larger organisms with increased motility—undesirable characteristics for early post-settlement recruits. On the other hand, rotifers present a more desirable option, since they are a renewable resource that can be nutritionally enriched whilst maintaining an appropriate size and motility [24]. Although rotifers must be ingested promptly to avoid the issue of retroconversion of DHA to 20:5n-3 (EPA) [25], they are commonly used as live prey in aquaculture [26,27], and have been successfully applied in laboratory trials investigating feeding in adult corals [28–31].

Therefore, this study aimed to evaluate the efficacy of three distinct heterotrophic feeding regimes (LC-PUFA-enriched rotifers (ROT), a novel, micro-bound diet rich in LC-PUFA (ATF), and unfiltered seawater, constituting a natural source of dissolved nutrients, suspended particulate matter, and plankton—which are potential coral food sources [32] (RAW), against a wholly phototrophic treatment (CTL), on the survival, growth, and nutritional composition of newly settled coral larvae over a 93 day experimental period. Four *Acropora* species (*A*. *hyacinthus*, *A*. *loripes*, *A*. *millepora*, and *A*. *tenuis*) were investigated in this study to gain a preliminary understanding of the feeding differences in closely related corals and assess the feasibility of a single feeding regime for multiple species in captivity.

## Materials and methods

### Broodstock and spawning

Gravid coral colonies (diameter: >20cm) of *A*. *hyacinthus*, *A*. *loripes*, *A*. *millepora*, and *A*. *tenuis* were collected from Trunk Reef (18° 18.173”S, 146° 52.153”E) in the Great Barrier Reef, Queensland, Australia between the 1st– 7^th^ of November, 2014 (Field collections were approved by the Great Barrier Reef Marine Park Authority: G12/35236.1). Corals were transferred to the National Sea Simulator facility (SeaSim) at The Australian Institute of Marine Science (AIMS), Townsville, Australia (16° 17.728"S, 145° 27.121"E) and placed in flow-through tanks under outdoor conditions of natural sunlight, at ~28°C until spawning (~15 colonies tank^-1^). All species spawned between the 10^th^ and 14^th^ of November, 2014. Gametes were collected from 6 parental colonies per species, fertilized, and the azooxanthellate larvae cultured at <500 larvae L^-1^ in flow-through tanks containing ultra-filtered seawater as per the methods of Negri and Heyward [33].

### Experimental design

At two days post-spawn, each larval species was settled separately onto aragonite coral plugs. These plugs were ‘pre-conditioned’ with crustose coralline algae (CCA), predominantly of the species *Titanoderma prototypum* for approx. 30 days in flow-through tanks (ultra-filtered seawater), at ~28°C, under outdoor conditions of natural sunlight through shade cloth. CCA is known to induce settlement and metamorphosis in scleractinian coral larvae [34], while simultaneously preventing pest algae and biofilm growth through allelopathy [35]. In most cases, ~5–15 individual recruits settled on each plug, with some gregarious settling (recruits settling together and fusing upon contact [8]) (1–6 fused colonies plug^-1^). Since recruit survivorship in *ex situ* facilities has been historically low [36,37], no attempt was made to obtain equal recruit numbers on each plug in order to maximize the sample size. This was then accounted for in the statistical analyses. Recruit numbers and surface areas were determined under light microscopy. After settlement, 41 plugs from each species were randomly distributed across 12 PVC trays (164 plugs tray^-1^). Trays were then placed into 12 49L tanks with replicate conditions including a 0.8 L^-1^ flow-through rate (1 x turn over hr^-1^) as well as a circulation pump to assist with water movement. Temperature and lighting remained constant at 26.8±0.1°C and ~150±8.0 μmol photons m^−2^s^−1^ (illumination period: 9.5h day^-1^, 1h ramp sunrise/sunset), respectively. For the first two weeks, an adult fragment from each species was included in all tanks to facilitate zooxanthellae uptake by the newly settled coral [2].

Three tanks were randomly assigned one of the four feeding regimes in a single-factorial design to assess recruit growth, survival, and total lipid, lipid class, and FA composition in response to each treatment. The four treatments were enriched rotifers (*Brachionus plicatilis*) (ROT), a novel, formulated diet developed at AIMS (ATF), unfiltered seawater (RAW) (sourced from Cleveland Bay, lat.: 19°155.83’S, long.: 146°88.116’E), and a control treatment of ultra-filtered seawater only (CTL). The CTL seawater was ultra-filtered to 0.04 μm (nominal size), removing most particulates. With the exception of RAW, the exogenous diet tanks were also supplied with CTL seawater. The RAW treatment was supplied continuously at the same flow rate as the CTL. The two exogenous diets were fed twice daily at 1000 and 1600 hrs. ROT were enriched for 24h in an emulsion rich in n-3 LC-PUFA (INVE Selco S.parkle), harvested daily and fed at 2.4 ROT ml^-1^, which represents a ‘high’ concentration of zooplankters observed on coral reefs [28,38] (~0.05 tank^-1^ DW). The ATF diet was prepared fresh daily, particulated to ≤40μm, suspended in ultrafiltered seawater, and fed out at 10 ml tank^-1^ (0.05g tank^-1^ DW) (commercial-in-confidence formulation, formulation details not provided). Circulation pumps were turned off during feeding for 20 minutes to facilitate capture efficiency and maximise feed consumption. Circulation pumps were then turned on briefly to evoke a ‘pulse’ effect, causing any settled feed to be resuspended, then left off for a further 20 minutes. Incoming water flow remained constant during feeding. Tanks were siphoned daily to remove remaining food and debris, while herbivorous snails (*Thalotia strigata*) were included in each tank to assist with algae and microfilm removal from tank walls.

### Coral sampling

The experiment duration was 93 days. A triplicate sample of coral in the planktonic larval stage was taken for each species two days post-spawn (pre-settlement) (~0.5g sample^-1^) and analysed for biochemical composition. Recruits were assessed for survival, growth (surface area to nearest mm^2^), and post-settlement fusion at three time points: experiment commencement (2 days post-settlement) (T0), T0 + 46 days (T1), T0 + 93 days (T2). Survival (%) was measured as (‘total recruits at T1 or T2’/ ‘total recruits at T0’) x 100. Surface area was measured using the software, Fiji ImageJ [39] (20 recruits species^-1^ tank^-1^ (60 recruits species^-1^ treatment^-1^)). Recruits were considered fused when tissue connection occurred between neighbouring recruits post-settlement. At T2, all recruits were removed from the plugs and frozen for subsequent proximate, lipid class, and FA analyses.

### Proximate, lipid class and fatty acid analysis

Samples were analysed for lipid and ash composition following standardised procedures described previously by Conlan et al. [40]. Briefly, lipids were extracted by cold extraction with dichloromethane:methanol (2:1), while ash was determined by incineration in a muffle furnace (C & L Fetlow, Model WIT, Blackburn, Victoria, Australia) at 450°C for 12 h. The ash content was then subtracted from the total dry weight to obtain ash free dry weight (AFDW) and permitted lipid quantification sans the calcium carbonate skeleton component. The exogenous diet total protein contents were measured using the Kjeldahl method (crude protein calculated as nitrogen×6.25) in an automated Kjeltech analyser (Tecator, Sweden). Due to very small sample size, total protein analysis was not possible for coral samples.

Lipid class analysis was determined using an Iatroscan MK 6s thin layer chromatography-flame ionisation detector (Mitsubishi Chemical Medience, Tokyo Japan) according to the method of Conlan et al. [40]. Briefly, each sample was spotted in duplicate on silica gel S5-chromarods (5 μm particle size) with lipid separation following a two-step elution sequence: 1) phosphatidylethanolamine (PE), phosphatidylcholine (PC) and lysophosphatidylchloline (LPC) elution was achieved in a dichloromethane/methanol/water (50:20:2, by volume) solvent system run to half height (~15 min); and 2) after air drying, wax ester (WAX), triacylglycerol (TAG), free fatty acid (FFA), 1,2-diacylglycerol (1,2DAG), and sterol (ST) elution was achieved in a hexane/diethyl ether/formic acid (60:15:1.5, by volume) solvent system run to full height (~30 min). Since glycolipids commonly elute with monoacylglycerols and pigments, including chlorophyll, the term “acetone mobile polar lipid” (AMPL) was used in the present study [41]. AMPL was quantified using the 1-monopalmitoyl glycerol standard (Sigma-Aldrich Co., USA), which has demonstrated a response that is intermediate between glycoglycerolipids and pigments [41].

Following initial lipid extraction, FA were esterified into methyl esters using an acid-catalysed methylation method and then analysed by gas chromatography as recently described in Conlan et al [40].

### Water quality analyses

Both the CTL and RAW seawater treatments were sampled weekly in duplicate for water quality analyses to identify the major differences between the unfiltered and ultrafiltered seawater. These analyses included five dissolved inorganic nutrients (5 DIN), namely NH_4_, PO_4_, NO_2_, NO_3_, and SiO_2_, as well as particulate organic carbon (POC) and nitrogen (PON), dissolved organic carbon (DOC), particulate phosphorus (PPO_4_), and the algal pigments, chlorophyll *a* and phaeophyll. Flow cytometry was also conducted to determine the amounts of bacteria-sized cells and virus-sized particles in the CTL and RAW seawater. In the laboratory, the samples were analysed following standard procedures [42].

## Statistical analysis

For each biometric, the n values were as follows: survival: n≥400 recruits, growth: n = 60 recruits, fused colonies: n≥400 recruits, biochemical analyses: n = 3 (all recruits in each replicate tank were pooled to obtain adequate sample mass). Data were analysed statistically using R software version 2.3.1 [43,44]. Untransformed water quality analyses data were analysed with a Welch two-sample t-test at a significance level of *p*<0.05. Due to non-normality and heteroscedasticity (detected via Shapiro-Wilk and Levene’s tests, respectively), as well as some negative values, all other data were transformed using a Yeo-Johnson power transformation (*caret* package [45]). Transformed data were then analysed using a one-way analysis of variance (ANOVA) for each parameter measured. Since the initial number of settled recruits on each plug may have influenced survival, growth, and fusion rates, these results were analysed with an analysis of covariance (ANCOVA), including the initial number of settled recruits as a covariate. Where statistical differences were detected, a TukeyHSD *post-hoc* test was employed at a significance level of *p*<0.05 (*agricolae* package [46]). Lipid class (% lipid) and FA profiles (% FA) were also analysed with one-way permutational ANOVAs (PERMANOVAs) using 999 permutations (vegan package [47]). Where differences were detected between treatments, similarity of percentages (SIMPER) analyses were conducted to determine which individual classes and FA drove the observed differences (vegan package [47]). These analyses were then visualized with a canonical analysis of principal coordinates (CAP) based on a discriminant analysis of 2 axes for lipid class, and 11 axes for FA (as determined by the classification success method of Anderson and Willis [48]), using a non-parametric Bray—Curtis similarity matrix (BiodiversityR package [49]). Ellipses show 95% confidence intervals for each treatment. Graphs were prepared using the ggplot2 package [50].

## Results

### Water quality analyses

Welch two-sample t-test results showed significantly higher amounts of PO_4_ (t = 1.82) and NO_2_ (t = 2.82), as well as NO_2_:NO_3_ (t = 1.98) in the RAW seawater relative to the CTL (*p*<0.05) (S1 Table). NH_4_, SiO_2_, POC, PON, DOC, and PPO_4_ levels were also higher in the RAW, although these were not significant. The algal pigments, chlorophyll *a* and phaeophyll were present in significantly higher concentrations in the RAW (0.15 ± 0.08 and 0.54 ± 0.34 μg L^-1^ respectively), with the CTL concentrations being negligible (≤0.01 μg L^-1^). The flow cytometry t-test results also showed significantly higher bacteria-sized cell (t = 10.8) and virus-sized particle (t = 5.92) quantities in the RAW treatment (*p*<0.01).

### Exogenous diet biochemical analyses

Both the ATF and ROT diets were high in moisture (>800 mg g sample^-1^) (Table 1). Total lipid and protein were higher in the ROT diet (9.41 ± 1.2 and 92.5 ± 2.12 mg g wet sample^-1^, respectively) compared to the ATF (5.66 ± 0.41 and 21.5 ± 0.14 mg g wet sample^-1^, respectively).

**Table 1:** Proximate and lipid class composition of ATF and ROT diets.

Proximate composition *(mg g sample*^*-1*^*)*	ATF	ROT
**Moisture**	892 ± 8.1	858 ± 3.21
**Lipid**	5.66 ± 0.41	9.41 ± 1.2
**Protein**	21.5 ± 0.14	92.5 ± 2.12
**Ash**	61.4 ± 0.13	13 ± 0.21
**Lipid class composition** *(mg g lipid*^*-1*^*)*		
**Wax ester**	90.8 ± 5.22	95 ± 6.97
**Triacylglycerol**	74.5 ± 10.9	60.2 ± 4.85
**Free fatty acid**	58.2 ± 4.73	46.5 ± 2.07
**1,2-diacylglycerol**	0 ± 0	0 ± 0
**Sterol**	80.4 ± 0.67	83.9 ± 8.29
**AMPL**	256 ± 6.52	280 ± 24.5
**Phosphatidylethanolamine**	149 ± 7.93	171 ± 9.53
**Phosphatidylcholine**	180 ± 7.65	202 ± 14.7
**Lysophosphatidylcholine**	111 ± 1.7	61.2 ± 6.12
**∑STORAGE**	223 ± 11.4	202 ± 4.19
**∑STRUCTURAL**	777 ± 11.4	798 ± 4.19
**STORAGE:STRUCTURAL**	0.29 ± 0.02	0.25 ± 0.01

Values are presented as means ± SEM.

For the lipid class composition (mg g lipid^-1^) (Table 1), the ATF diet contained higher storage lipid compared to the ROT (223±11.4 and 202±4.19 mg g lipid^-1^, respectively), largely in the form of TAG and FFA, while the ROT diet was richer in the phospholipids, PE, PC, and LPC.

The ROT diet contained a higher total FA concentration relative to the ATF (666 ± 38.2 and 560 ± 19.4 mg g lipid^-1^, respectively) (Table 2). The ATF diet was dominated by saturated fatty acids (SFA) and PUFA (292±9.27 and 200±8.48 mg g lipid^-1^, respectively), largely in the form of 16:0 and 22:6n-3 (DHA). The ROT diet was dominated by monounsaturated fatty acids (MUFA) (377 ± 30.7 mg g lipid^-1^), largely in the form of 16:1n-7 and 18:1n-9.

**Table 2:** Fatty acid composition of ATF and ROT diets.

*(mg g lipid*^*-1*^*)*	ATF	ROT
**14:0**	46 ± 2.12	10.3 ± 0.49
**16:0**	181 ± 7.28	84.1 ± 3.3
**18:0**	42.5 ± 0.7	35.6 ± 1.65
**∑SFA**	**292 ± 9.27**	**145 ± 5.64**
**16:1n-7**	8.63 ± 0.38	113 ± 13.1
**18:1n-9**	42.6 ± 0.7	176 ± 15
**20:1n-9**	1.62 ± 0.03	22.3 ± 1.53
**20:1n-11**	2.31 ± 0.11	4.24 ± 0.44
**∑MUFA**	**67.6 ± 1.82**	**377 ± 30.7**
**18:2n-6**	14 ± 0.12	57.8 ± 2.43
**18:3n-6**	1.3 ± 0.08	0.74 ± 0.06
**20:4n-6**	7.45 ± 0.07	5.23 ± 0.37
**20:5n-3**	8.29 ± 0.27	16.1 ± 0.83
**22:6n-3**	116 ± 5.48	11.2 ± 0.75
**∑PUFA**	**200 ± 8.48**	**143 ± 6.08**
**TOTAL**	**560 ± 19.4**	**666 ± 38.2**
**∑n-3 PUFA**	136 ± 6.34	64.2 ± 2.57
**∑n-6 PUFA**	134 ± 6.34	50.8 ± 1.93
**∑n-3 LC PUFA**	64 ± 2.11	77.9 ± 3.18
**∑n-6 LC PUFA**	47.9 ± 2.13	13.2 ± 0.6
**n-3:n-6**	2.12 ± 0.03	0.82 ± 0
**LC n-3:LC n-6**	2.8 ± 0.01	3.85 ± 0.11
**EPA:DHA**	0.07 ± 0	1.43 ± 0.05
**EPA:ARA**	1.11 ± 0.03	3.08 ± 0.1

Values are presented as means ± SEM.

### Effect of feeding regimes on recruit survival and growth

**Fig 1:**
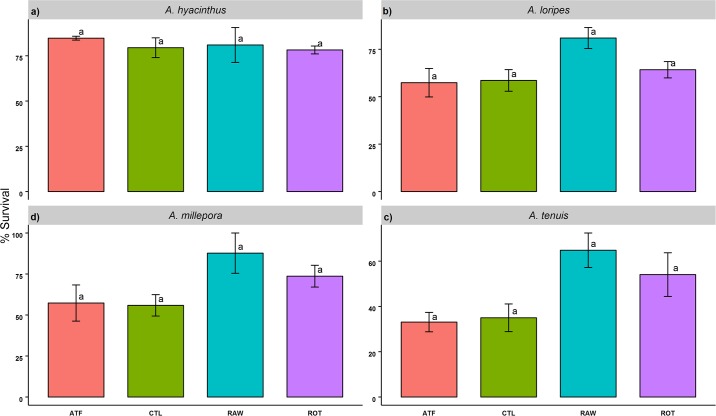
Survival of *Acropora* recruits fed four different feeding regimes over 93 days (% survival). Values are presented as means ± SEM. Letters not in common in each plot denote significant differences, as determined with TukeyHSD *post-hoc* tests (*p*<0.05).

After 93 days, *A*. *hyacinthus* showed consistently high survival across treatments (>78%) (Fig 1). Meanwhile, the RAW treatment recorded the highest survival for *A*. *loripes*, *A*. *millepora*, and *A*. *tenuis*, although this was not statistically significant. *A*. *tenuis* had the lowest survival rates of all species, particularly in the ATF and CTL treatments (33.1 ± 3.71% and 35 ± 5.29%, respectively).

**Fig 2:**
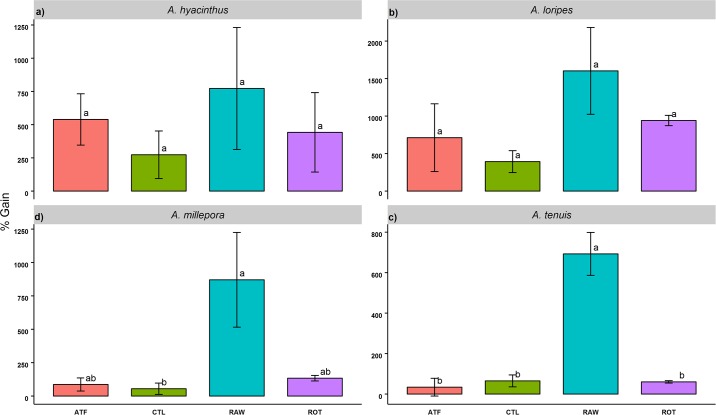
Growth of *Acropora* recruits fed four different feeding regimes over 93 days (% gain). Values are presented as means ± SEM. Letters not in common in each plot denote significant differences, as determined with TukeyHSD *post-hoc* tests (*p*<0.05).

Growth was highest in the RAW treatment for all species (% surface area gain) (Fig 2). The CTL treatment recorded the lowest growth for *A*. *hyacinthus*, *A*. *loripes*, and *A*. *millepora*, while the ATF treatment was lowest for *A*. *tenuis*. Reduced growth occurred between T1-T2 in the CTL treatment for several *A*. *millepora* and *A*. *tenuis* individuals, causing a mean growth reduction in the former (-9.15 ± 11.3%), and a low mean gain in the latter (0.68 ± 15.1%) (S3 Table). One-way ANCOVA results yielded significant variation in growth among treatments for *A*. *millepora* (F(4, 6) = 5.206, *p*<0.05) and *A*. *tenuis* (F(4, 6) = 15.99, *p*<0.01). TukeyHSD *post-hoc* analyses showed that the RAW treatments differed significantly from the CTL for *A*. *millepora*, while for *A*. *tenuis*, the RAW differed significantly from all other treatments (*p*<0.05).

**Fig 3:**
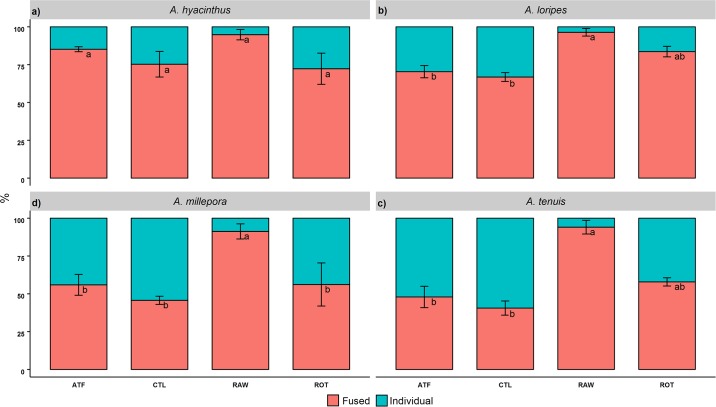
Proportion of fused: individual *Acropora* recruits fed four different feeding regimes over 93 days (%). Values are presented as means ± SEM. Letters not in common in each plot denote significant differences, as determined with TukeyHSD *post-hoc* tests (*p*<0.05).

The RAW treatment exhibited the highest proportion of fused colonies for all species (>90% fused) (Fig 3). In addition to the reduced growth, reduced fusion rates occurred between T1-T2 for *A*. *millepora* and *A*. *tenuis* in the CTL treatment (-12% and -9%, respectively). The CTL treatment had the lowest fusion rates for *A*. *loripes*, *A*. *millepora*, and *A*. *tenuis*, while for *A*. *hyacinthus*, the ROT treatment exhibited the least fused recruits. One-way ANCOVA results showed significant variation in fusion among treatments for *A*. *loripes* (F(3, 7) = 7.86, *p*<0.05), *A*. *millepora* (F(5, 5) = 46.5, *p*<0.01) and *A*. *tenuis* (F(5, 5) = 10.7, *p*<0.01). TukeyHSD *post-hoc* analyses showed that the RAW treatment was significantly higher compared to the ATF and CTL for *A*. *loripes* and *A*. *tenuis*, while for *A*. *millepora*, the RAW was significantly higher than all other treatments (*p*<0.05).

### Larvae and recruit nutrition

#### Proximate and lipid class compositions

Total lipid content was typically high in the larvae, ranging from 857 mg g AFDW^-1^ for *A*. *tenuis* and 997 mg g AFDW^-1^ in *A*. *millepora* (S1 Fig).

**Fig 4:**
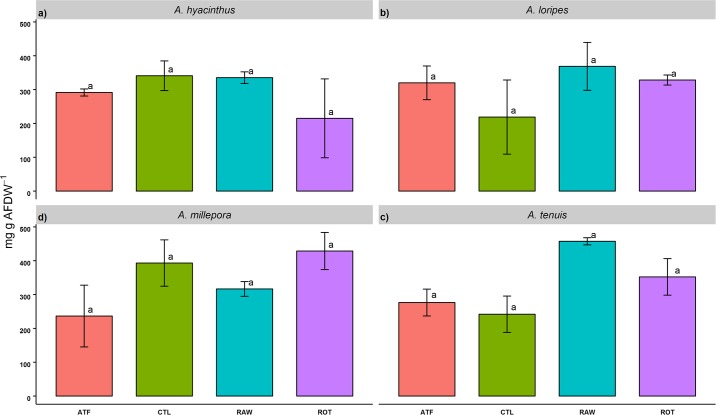
Total lipid concentration of *Acropora* recruits fed four different feeding regimes over 93 days (mg g AFDW^-1^). Values are presented as means ± SEM. Letters not in common in each plot denote significant differences, as determined with TukeyHSD *post-hoc* tests (*p*<0.05).

In the recruits, feeding regime had no significant effect on total lipid content for all species (mg g AFDW^-1^) (Fig 4).

**Fig 5:**
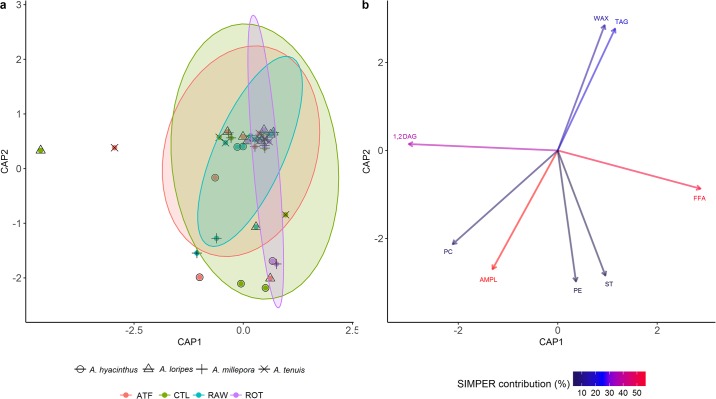
Canonical analysis of principal components (CAP) a) score plot and b) biplot, showing overall lipid class profile (% lipid) of *Acropora* recruits fed four different feeding regimes over 90 days. a) Ellipses show 95% confidence intervals for each treatment. b) Vectors show top ten individual classes contributing to the overall variance between treatments. Colour gradient shows percentage contribution to overall variance, obtained with SIMPER analysis.

Lipid class composition was variable within the recruits (S3 Fig) and this was reflected in the PERMANOVA, which showed no significant differences in the overall lipid class profiles between any treatments. This is graphically illustrated in the CAP score plot (Fig 5a), which shows overlapping 95% confidence intervals for all treatments. The biplot shows that the first axes (CAP1) loaded 1,2DAG on the negative side, and FFA on the positive side (Fig 5b), and these largely influenced the scoring of the ATF and CTL treatments. The second axis (CAP2) shows grouping of the storage lipids, WAX and TAG on the positive side, and the structural lipids ST, AMPL, PC, and PE on the negative side. While the score plots show that all treatments were dispersed along CAP2, this was most pronounced for the RAW and ROT treatments.

#### Fatty acid composition

Larvae of all species consisted predominantly of SFA, largely in the form of 16:0 (~80–110 mg g lipid^-1^), followed by 10:0, 14:0, 16:1n-7, 18:1n-9, and 18:3n-6 (S6 Table).

Several changes were notable in the quantitative FA concentrations (mg g lipid^-1^) between the larval and recruit stages, regardless of feeding regime. For example, 10:0 was virtually depleted from the larval stage to the recruit stage, while 12:0 and 22:6n-3 (DHA) underwent substantial increases (S7–S10 Tables). These quantitative changes caused a reshuffling of the qualitative concentrations (% FA). Of particular note was the total PUFA, which ranged from ~19–24%FA in the larvae, and ~22–30%FA in the recruits.

Interestingly, the proportions of several individual long-chain PUFA (LC-PUFA) in recruits fed the ATF treatment did not increase significantly from the larvae, whereas most other treatments did. For instance, one-way ANOVA and subsequent TukeyHSD *post-hoc* results revealed significant increases in 20:5n-3 (EPA) from the larval stage in all treatments except the ATF for *A*. *hyacinthus* (F(4, 9) = 3.87, *p*<0.05), *A*. *loripes* (F(4, 9) = 5.83, *p*<0.05), and *A*. *tenuis* (F(4, 9) = 18.3, *p*<0.01) (% FA). Additionally, 20:4n-6 (ARA) increased significantly from the larvae to the recruits in the CTL and RAW treatments, but not the ATF treatment for *A*. *hyacinthus* (F(4, 9) = 76.1, *p*<0.001), *A*. *loripes* (F(4, 9) = 30.7, *p*<0.001), and *A*. *tenuis* (F(4, 9) = 4.85, *p*<0.05) *(*% FA).

**Fig 6:**
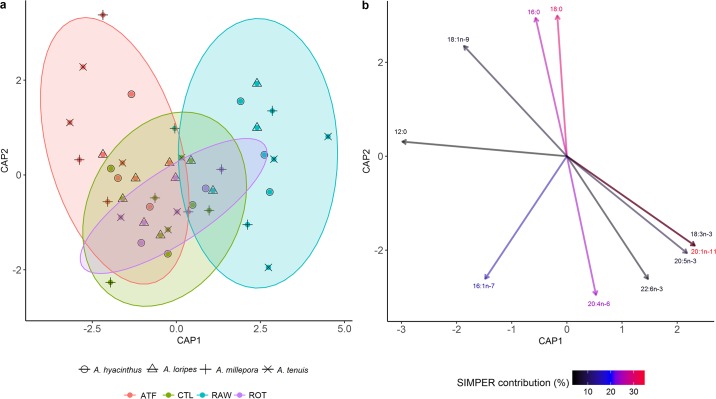
Canonical analysis of principal components (CAP) a) score plot and b) biplot, showing overall fatty acid profile (% fatty acids) of *Acropora* recruits fed four different feeding regimes over 90 days. a) Ellipses show 95% confidence intervals for each treatment. b) Vectors show top ten individual fatty acids contributing to the overall variance between treatments. Colour gradient shows percentage contribution to overall variance, obtained with SIMPER analysis.

The one-way PERMANOVA for the overall fatty acid profiles of the recruits showed significant differences between the RAW and CTL treatments (r = 0.20, *p*<0.01), and the RAW and ATF treatments for all species (r = 0.29, *p*<0.001) (%FA). This is graphically demonstrated in the CAP score plot, which showed that the ATF and CTL treatments were similar to each other, yet clearly separated from the RAW treatment (Fig 6a). The CAP biplot (Fig 6b) shows that the separation of the ATF treatment is largely driven by the SFA, 16:0, 18:0, and 12:0, as well as the MUFA, 18:1n-9. Meanwhile the CTL treatment is largely influenced by 12:0 and the MUFA, 16:1n-7. On the other hand, the RAW treatment separation is largely driven by the MUFA, 20:1n-11, and several PUFAs, namely ARA, EPA, DHA, and 18:3n-3.

Interestingly, despite high 18:1n-9 levels in the ROT diet (Table 2), elevated levels were not evident in the recruits fed this treatment (S7–S10 Tables). Similarly, despite the high PUFA levels in the ATF diet, particularly DHA, significantly higher levels were not reflected in the recruits for this treatment (S7–S10 Tables).

## Discussion

Understanding the nutritional requirements of coral recruits, particularly in relation to lipids and their constituents, gives critical insight into the biochemical processes driving recruit survival, growth, and overall health. This is fundamental in informing sexual propagation procedures and furthering the prospects of closed coral life cycles in captivity through optimised feeding strategies. Here, we provide a unique insight into the nutritional composition of captive coral recruits and highlight the degree of nutritional augmentation in response to different feeding regimes, generating new trajectories for coral nutrition research.

Survival across all species (~33–87% over 3 months) was comparable to previously recorded rates of scleractinian recruits reared *ex situ* (~10–85% over 3–12 months) [2,3,51]. Generally, survival was higher in the heterotrophic feeding regimes from T1-T2. This likely reflects a life phase shift to a more robust juvenile stage, as the first 4–6 weeks post-settlement constitute the most vulnerable stage in a coral’s benthic phase, largely due to sub-optimal photosynthetic capacities resulting from settlement preference for cryptic, low light areas, and low zooxanthellae populations [2,52,53]. Thus, heterotrophic feeding during this period is critical to provide an adequate energetic substrate to facilitate basal metabolic requirements, survival, and early growth prior to the establishment of strong symbioses.

Comparing treatments, the RAW promoted the highest survival for *A*. *loripes*, *A*. *millepora*, and *A*. *tenuis*. This was largely mirrored in the results achieved for growth and fusion, which were also highest in the RAW treatment for all species. Coral fusion describes neighbouring recruits growing toward each other and fusing upon contact, forming a single, larger colony [8]. Post-settlement fusion and colonial growth enables recruits to grow faster than single polyps, as they possess larger energy stores and occupy increased space for enhanced photosynthesis and exogenous food capture—leading to greater resource acquisition [54]. Since size is an important survival determinant in scleractinian recruits [2], increased heterotrophy can enhance post-settlement fusion and accelerate corals through the initial vulnerable phase toward a juvenile size-refuge. Indeed, survival rates in scleractinian corals have been shown to increase significantly with size and fusion [8]. This provides a crucial head-start in recruit ontogeny, and can hence reduce the time and costs required for coral culture [2].

While it is difficult to directly compare the present results with those published previously, the RAW treatment clearly provided considerable advantages over previous heterotrophic approaches. For example, *A*. *tenuis* recruits fed unenriched *Artemia* (3750 nauplii L^-1^) in a previously published study [2] obtained a lower total surface area (~9.5mm^2^) compared to *A*. *tenuis* subjected to the RAW treatment in the present study (16.8 ± 1.81 mm^2^), over an equivalent experimental period (three months). However, considering the results of Petersen et al [2] are limited to survival and growth, further comparisons of nutritional integrity and fortification cannot be made. None-the-less, the physiological results of the RAW treatment indicate that future feeding regimes should seek to emulate the characteristics of unfiltered, ‘raw’ seawater in order to accelerate recruit growth toward a juvenile size-refuge.

The lowest survival, growth, and fusion rates were recorded in the CTL treatment for most species. This included reduced growth in *A*. *millepora*, as well as decreases in fused colonies for both *A*. *millepora*, and *A*. *tenuis* due to partial colony mortalities, resulting in a single polyp remaining alive (pers. obs.). Poor survival and growth has previously been shown in unfed scleractinian recruits [2,55], and these results further demonstrate the inhibitory effect of a wholly phototrophic approach on recruit development, prolonging the vulnerable, post-settlement phase. This delayed development is likely due to limited structural resources, such as phospholipids and amino acids, in photosynthetically-derived carbon [56]. These nutrients are critical for organ development and skeletal deposition, and must largely be obtained through heterotrophy [56].

Interestingly, the superior growth and fusion results for the RAW treatment were not reflected in the total lipid content, indicating coral recruits maintain lipid to a constant level, regardless of diet. Previous reports on adult corals suggest that when photosynthesis is maximal, heterotrophic nutrients are directed toward tissue and skeletal growth rather than accumulated lipid reserves [14], since much of the coral’s energetic requirement is met by phototrophy. This agrees with the RAW treatment’s physical biometrics, which suggest surplus lipids were directed toward growth.

Similarly, despite the greater physiological results elicited by the RAW treatment, no trends were evident in the lipid class composition, either between treatments or species. Furthermore, high variability was evident between sample replicates, shrouding treatment effects within-species. In the RAW treatment, *A*. *hyacinthus* and *A*. *tenuis* contained markedly higher structural lipid proportions, largely in the form of AMPL, PE, and PC, while *A*. *millepora* was dominated by storage lipids, largely in the form of TAG and FFA. *A*. *loripes*, in contrast, contained similar storage and structural lipid proportions in most treatments. Genus-specific differences in lipid class composition have been recorded previously [13,57]. However, the present study demonstrates vast differences even within a single genus.

In the planulae larvae, the FA composition was generally similar between species. SFA and MUFA predominated, mainly as 10:0, 14:0, and 16:0, and 16:1n-7, 18:1n-9, and 20:1n-9, respectively, which agrees with previous reports for acroporid eggs [23,53]. These FA are readily catabolised and thus provide major energy sources during lecithotrophic development, providing energy for swimming and the energetically-expensive metamorphosis process [23,26]. Notably, 10:0 was significantly diminished in the recruits, suggesting its importance during larval development. Medium-chain FA, such as 10:0, are readily utilised, and constitute an important and easily accessible energy source in natal nutrition [58].

In most cases, the total FA concentration in the larval stage (~300 mg g lipid^-1^) decreased in the recruit stage (~200 mg g lipid^-1^), which is likely attributable to lipid class re-compartmentalization to meet the vastly different cellular requirements of coral recruits post-metamorphosis. Despite this, the quantitative amounts of EPA generally remained similar or actually increased in the recruit stage. This caused an approximate two-fold increase in the qualitative proportions of EPA in the recruits, demonstrating a major restructuring of the FA composition in the benthic stage, and potentially the sparing of EPA. It is well recognised that PUFA are preferentially spared over SFA and MUFA to preserve essential biological membrane components during lecithotrophy [59,60].

DHA also increased significantly in the recruits, regardless of treatment. This conforms to previous findings, which suggest a negligible role of DHA during the coral larval phase [23], yet a significant role in the benthic phase [61]. Low DHA concentrations in coral larvae may reflect the more complex oxidation process required for DHA catabolism compared to EPA [62]. Moreover, DHA is a key component in growth and development, including cell membrane formation [59,63], which occur post-settlement.

There was also a marked increase in ARA concentrations from larvae to recruits. Both EPA and ARA are eicosanoid precursors, which are critical for numerous physiological processes, including pigmentation [22], which also occurs post-settlement. Furthermore, EPA, DHA, and ARA are involved in immunity and maintaining cell membrane structure and function [59,61]. As such, the increases in these FA in the benthic stage reflect their critical roles in adult life processes. Therefore, the strong influence of these FA on the separation of the RAW treatment from all other treatments indicates advanced physiological development of recruits in this treatment, further emphasising the importance of adequate PUFA levels in early recruit nutrition.

On the other hand, low PUFA relative to SFA, which occurred in the ATF and CTL treatments, has been shown in scleractinian corals following stress [61,64]. Nutritional stress affects corals’ ability to photosynthesise and assimilate inorganic nutrients, which can impact both the symbiont and host FA profiles in a number of complex ways [64]. Indeed, elevated levels of the individual FA, 16:0, 18:0, and 18:1n-9 relative to PUFA have been suggested to be indicative of stress. Tellingly, these were the major FA contributing to the dissimilarity between the ATF and CTL treatments compared to the RAW. Importantly, 18:1n-9 was present in relatively low concentrations in the ATF diet (Table 2), supporting the theory that elevated levels in this treatment resulted from stress. In addition, several FA in the ATF treatment did not undergo significant changes between the larval and recruit stages (% FA), suggesting a slower development rate towards juvenility.

The adverse response elicited by the ATF treatment may be attributable to several factors, including excessive dietary lipid or protein, which have previously shown reduced survival and growth in marine larvae, due to a nutritional overload of the underdeveloped digestive system [65]. As such, a nutritionally dilute form of the ATF diet may prove more beneficial during the early recruit stages. However, throughout ontogeny, improvements may be expected in recruit metabolism capabilities [60], which may explain the improved growth and survival results in the ATF treatment during the second half of the experiment (S2–S4 Tables). Thus, the ATF treatment in its current form may be better suited to adult corals, which are known to consume numerous food sources, including lipid-rich zooplankton [16].

The ROT treatment’s performance was generally an improvement on the ATF and CTL, yet inferior to the RAW treatment. Although the ROT diet was higher in lipid and protein than the ATF, its greater performance may be ascribable to the introduction of digestive enzymes from the live prey, facilitating the underdeveloped coral digestive system [60]. Interestingly, the major FA present in the ROT treatment, particularly 18:1n-9 and 16:1n-7, were not reflected in the recruits, suggesting low consumption, or their active metabolism. Indeed, zooplankton capture efficiency has been shown as limited in some corals, particularly under high flow conditions [66], which may be relatable to the feeding capacity of early-stage recruits [16].

On the other hand, a preference for phytoplankton over motile zooplankton has been recorded in some corals [66]. Thus, the RAW treatment’s improved performance may be attributable to preferential phytoplankton consumption by recruits. This is supported by the flow cytometry, chlorophyll, and phaeophyll results in the water quality analyses for the RAW treatment (S1 Table), which were significantly higher than the CTL seawater (which was also supplied to the two exogenous diets). Moreover, the significantly higher bacteria-sized cell concentration in the RAW treatment may implicate bacteria as an additional nutrition source, which has previously been suggested in scleractinian corals [16].

Additionally, water quality analyses showed significantly lower dissolved nitrogen and phosphate levels in the CTL in comparison to the RAW treatment. Despite the naturally oligotrophic conditions of coral reefs, low nutrient levels are not necessarily optimal for coral physiological performance, whereas slightly elevated concentrations have shown increased growth and photosynthetic efficiency [67], since zooxanthellae are known to assimilate and concentrate dissolved nutrients and transport them to the host for incorporation into vital organic molecules including DNA, RNA, proteins, and phospholipids [56]. Therefore, the RAW treatment’s superior performance may also be ascribable to increased availability of dissolved inorganic nutrients. In either case, nutrient acquisition in the RAW treatment, either from planktonic food or direct absorption from the water column, clearly promotes increased survival, growth, and development of acroporid recruits, as well as improved nutritional profiles. Due to the potential for bacteria and pathogen introduction, as well as seasonal and locational fluctuations in salinity and nutrient loads, unfiltered, ‘raw’ seawater may not present a feasible option for large-scale coral culture. However, these results can serve as foundational guidelines for the nutritional requirements of acroporid recruits, and future investigations should examine the RAW treatment’s beneficial characteristics, including dissolved nutrients and phytoplankton, to inform future dietary formulations of optimised nutrition, consistent quality, and minimal time and labour costs.

## Supporting information

S1 Fig Total lipid concentration of planktonic larvae for each *Acropora* species (mg g AFDW^-1^). (DOCX)

S2 Fig Lipid class composition of planktonic larvae for each *Acropora* species (mg g lipid^-1^). (DOCX)

S3 Fig Effect of different feeding regimes on the lipid class composition of *Acropora* recruits after 93 days (mg g lipid^-1^). (DOCX)

S1 Table Water quality analyses of CTL and RAW seawater. (DOCX)

S2 Table Effect of different feeding regimes on the survival of *Acropora* recruits (% survivors). (DOCX)

S3 Table Effect of different feeding regimes on the surface area gain of *Acropora* recruits (% gain). (DOCX)

S4 Table Effect of different feeding regimes on the proportion of fused *Acropora* recruits (% fused). (DOCX)

S5 Table Effect of different feeding regimes on the total lipid and ash composition of *Acropora* recruits after 93 days. (DOCX)

S6 Table Fatty acid composition of planktonic larvae for each *Acropora* species (mg g lipid^-1^ and % lipid). (DOCX)

S7 Table Effect of different feeding regimes on the fatty acid composition of *Acropora hyacinthus* recruits after 93 days (mg g lipid^-1^ and % lipid). (DOCX)

S8 Table Effect of different feeding regimes on the fatty acid composition of *Acropora loripes* recruits after 93 days (mg g lipid^-1^ and % lipid). (DOCX)

S9 Table Effect of different feeding regimes on the fatty acid composition of *Acropora millepora* recruits after 93 days (mg g lipid^-1^ and % lipid). (DOCX)

S10 Table Effect of different feeding regimes on the fatty acid composition of *Acropora tenuis* recruits after 93 days (mg g lipid^-1^ and % lipid). (DOCX)
