# Supplementary material for: Influence of different feeding regimes on the survival, growth, and biochemical composition of Acropora coral recruits
Source: PLoS One. 2017 Nov 28;12(11):e0188568. doi: 10.1371/journal.pone.0188568 (PMC5705105; doi:10.1371/journal.pone.0188568)
Supplement: S2 Fig — (DOCX) [file pone.0188568.s002.docx]

##
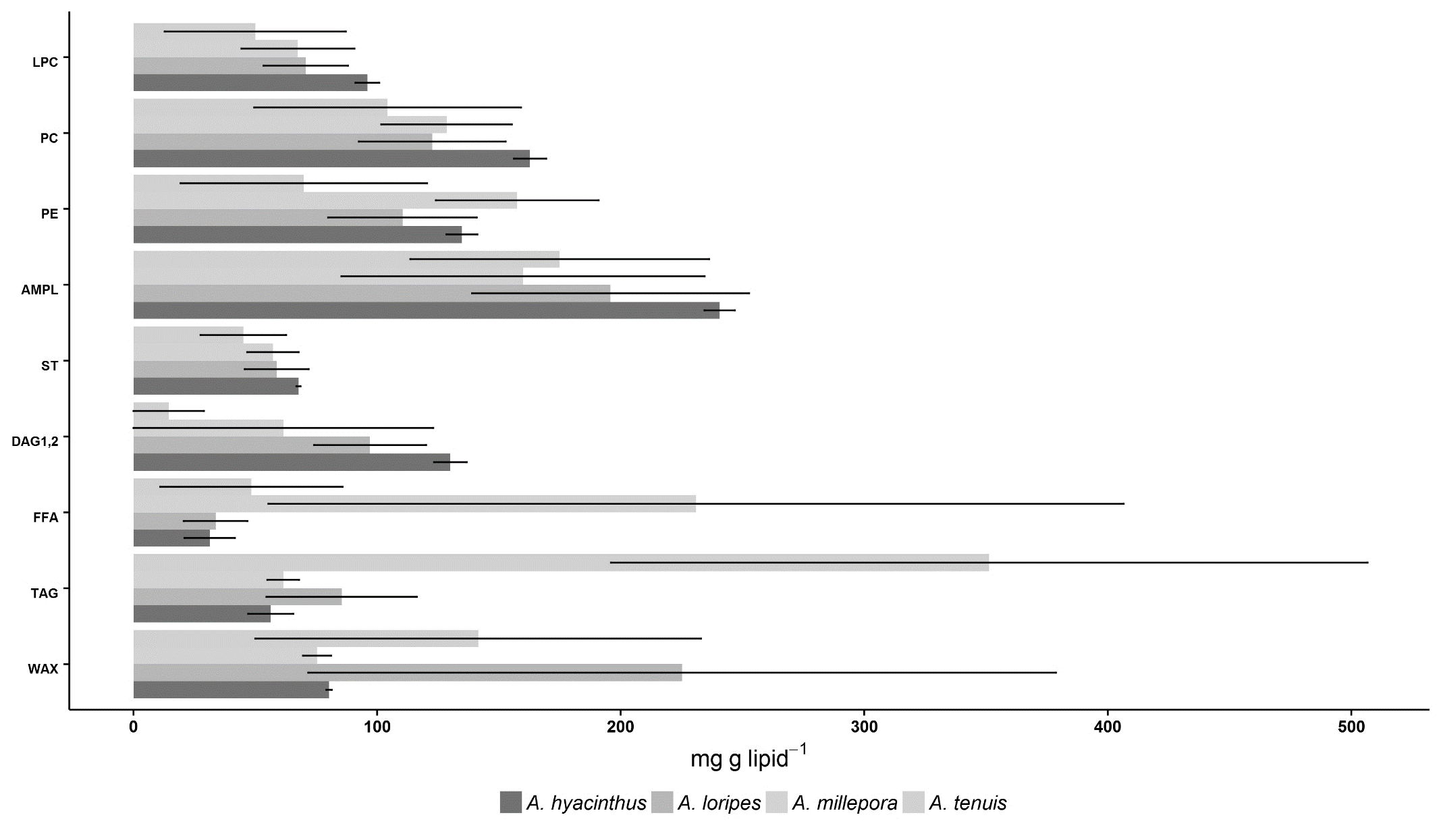
S2 Fig. Lipid class composition of planktonic larvae for each *Acropora* species (mg g lipid^-1^)

Values are presented as means ± SEM. Values in the same group that do not share the same letter are significantly different (P<0.05).
