# Supplementary material for: Influence of different feeding regimes on the survival, growth, and biochemical composition of Acropora coral recruits
Source: PLoS One. 2017 Nov 28;12(11):e0188568. doi: 10.1371/journal.pone.0188568 (PMC5705105; doi:10.1371/journal.pone.0188568)
Supplement: S1 Table — (DOCX) [file pone.0188568.s004.docx]

##### S1 Table Water quality analyses of CTL and RAW seawater

| **DIN** *(µmol L^-1^)* | **CTL** | **RAW** |
| --- | --- | --- |
| **NH_4_** | 1.13 ± 0.33^a^ | 1.33 ± 0.46^a^ |
| **NO_3_** | 1.22 ± 0.12^b^ | 6 ± 3.01^a^ |
| **NO_2_** | 0.07 ± 0.01^b^ | 0.19 ± 0.07^a^ |
| **PO_4_** | 0.13 ± 0.02^b^ | 0.33 ± 0.12^a^ |
| **SiO_2_** | 6.8 ± 1.94^a^ | 7.86 ± 1.86^a^ |
| **POC** *(µg L^-1^)* | 113 ± 89.4^a^ | 167 ± 182^a^ |
| **PON** *(µg L^-1^)* | 22.5 ± 14.7^a^ | 26.2 ± 24.3^a^ |
| **C:N** | 4.35 ± 1.18^a^ | 5.34 ± 1.47^a^ |
| **DOC** *(mg L^-1^)* | 0.94 ± 0.05^a^ | 0.98 ± 0.08^a^ |
| **POPh** *(µM)* | 7.52 ± 4.38^a^ | 10.9 ± 5.73^a^ |
| **Chl *a*** *(µg L^-1^)* | 0.01 ± 0.02^b^ | 0.15 ± 0.08^a^ |
| **Phaeo** *(µg L^-1^)* | 0.01 ± 0.02^b^ | 0.54 ± 0.34^a^ |
| **Bacteria-sized cells** *(x10^4^ ml^-1^)* | 33.6 ± 6.5^b^ | 126 ± 19^a^ |
| **Virus-sized cells (***x10^4^ ml^-1^)* | 45.2 ± 11.2^b^ | 332 ± 113^a^ |

Values are presented as means ± SEM. Values in the same row that do not share the same superscripts are significantly different (*P*<0.05).
