# Supplementary material for: Influence of different feeding regimes on the survival, growth, and biochemical composition of Acropora coral recruits
Source: PLoS One. 2017 Nov 28;12(11):e0188568. doi: 10.1371/journal.pone.0188568 (PMC5705105; doi:10.1371/journal.pone.0188568)
Supplement: S2 Table — (DOCX) [file pone.0188568.s005.docx]

##### S2 Table

##### Effect of different feeding regimes on the survival of *Acropora* recruits (% survivors). T0-T1: experiment commencement – 46 days, T1-T2: 46 days – 93 days, T0-T2: experiment commencement – 93 days.

| **Species** | **Time** | **ATF** | **CTL** | **RAW** | **ROT** |
| --- | --- | --- | --- | --- | --- |
| ***A. hyacinthus*** | **T0-T1** | 86.2 ± 1.3^a^ | 83.8 ± 7.04^a^ | 81 ± 6.81^a^ | 78.2 ± 1.54^a^ |
|  | **T1-T2** | 98.3 ± 1.43^a^ | 95.3 ± 2.35^a^ | 100 ± 0^a^ | 100 ± 0^a^ |
|  | **T0-T2** | 84.7 ± 0.93^a^ | 79.5 ± 4.73^a^ | 81 ± 6.81^a^ | 78.2 ± 1.54^a^ |
|  | | | | | |
| ***A. loripes*** | **T0-T1** | 66.9 ± 4.49^b^ | 82.5 ± 3.42^a^ | 80.9 ± 3.91^a^ | 67.1 ± 1.02^b^ |
|  | **T1-T2** | 86.9 ± 11.1^a^ | 71.8 ± 8.41^a^ | 100 ± 0^a^ | 95.6 ± 3.08^a^ |
|  | **T0-T2** | 57.4 ± 6.49^a^ | 58.6 ± 4.94^a^ | 80.9 ± 3.91^a^ | 64.2 ± 3.04^a^ |
|  | | | | | |
| ***A. millepora*** | **T0-T1** | 75.2 ± 4.74^b^ | 80.1 ± 2.94^ab^ | 92.9 ± 5.02^a^ | 82.6 ± 1.62^ab^ |
|  | **T1-T2** | 75.2 ± 9.41^a^ | 69.3 ± 4.81^a^ | 94 ± 4.27^a^ | 89.1 ± 3.95^a^ |
|  | **T0-T2** | 57.3 ± 9.55^a^ | 55.9 ± 5.66^a^ | 87.7 ± 8.68^a^ | 73.7 ± 4.7^a^ |
|  | | | | | |
| ***A. tenuis*** | **T0-T1** | 55.5 ± 3.57^a^ | 58.9 ± 6.9^a^ | 70 ± 1.73^a^ | 67 ± 4.67^a^ |
|  | **T1-T2** | 61.5 ± 11.4^a^ | 59.4 ± 4.51^a^ | 92.3 ± 5.41^a^ | 80 ± 4.59^a^ |
|  | **T0-T2** | 33.1 ± 3.71^a^ | 35 ± 5.29^a^ | 64.8 ± 5.38^a^ | 54.1 ± 6.82^a^ |

Values are presented as means ± SEM. Values in the same row that do not share the same superscripts are significantly different (*P*<0.05).
