# Supplementary material for: Influence of different feeding regimes on the survival, growth, and biochemical composition of Acropora coral recruits
Source: PLoS One. 2017 Nov 28;12(11):e0188568. doi: 10.1371/journal.pone.0188568 (PMC5705105; doi:10.1371/journal.pone.0188568)
Supplement: S3 Table — (DOCX) [file pone.0188568.s006.docx]

##### S3 Table Effect of different feeding regimes on the surface area gain of *Acropora* recruits (% gain). T0-T1: experiment commencement – 46 days, T1-T2: 46 days – 93 days, T0-T2: experiment commencement – 93 days.

| **Species** | **Time** | **ATF** | **CTL** | **RAW** | **ROT** |
| --- | --- | --- | --- | --- | --- |
| ***A. hyacinthus*** | **T0-T1** | 78.5 ± 44^a^ | 112 ± 41.8^a^ | 189 ± 102^a^ | 93.5 ± 22.3^a^ |
|  | **T1-T2** | 289 ± 85.5^a^ | 57.8 ± 37.2^a^ | 191 ± 51.1^a^ | 162 ± 79^a^ |
|  | **T0-T2** | 539 ± 167^a^ | 273 ± 155^a^ | 772 ± 397^a^ | 442 ± 211^a^ |
|  | | | | | |
| ***A. loripes*** | **T0-T1** | 91.9 ± 5.78^b^ | 107 ± 7.89^ab^ | 246 ± 36.8^a^ | 274 ± 14.2^a^ |
|  | **T1-T2** | 312 ± 191^a^ | 136 ± 54.8^a^ | 372 ± 109^a^ | 178 ± 2.43^a^ |
|  | **T0-T2** | 712 ± 391^a^ | 393 ± 127^a^ | 1600 ± 501^a^ | 941 ± 48.6^a^ |
|  | | | | | |
| ***A. millepora*** | **T0-T1** | 9.66 ± 21^a^ | 69.6 ± 28.3^a^ | 129 ± 40.4^a^ | 68.3 ± 20.9^a^ |
|  | **T1-T2** | 66.8 ± 9.76^ab^ | -9.15 ± 11.3^c^ | 299 ± 57.9^a^ | 41.1 ± 8.81^bc^ |
|  | **T0-T2** | 86.6 ± 42.6^ab^ | 54.7 ± 36.7^b^ | 870 ± 307^a^ | 134 ± 14.6^ab^ |
|  | | | | | |
| ***A. tenuis*** | **T0-T1** | 10.1 ± 6.7^b^ | 58.3 ± 18.2^a^ | 91.7 ± 32.6^a^ | 65.3 ± 20.6^a^ |
|  | **T1-T2** | 20.6 ± 31^b^ | 3.34 ± 5.4^b^ | 334 ± 71.7^a^ | 0.68 ± 15.1^b^ |
|  | **T0-T2** | 34.1 ± 38^b^ | 65.1 ± 25.5^b^ | 693 ± 91.8^a^ | 60.2 ± 4.2^b^ |

Values are presented as means ± SEM. Values in the same row that do not share the same superscripts are significantly different (*P*<0.05).
