# Supplementary material for: Influence of different feeding regimes on the survival, growth, and biochemical composition of Acropora coral recruits
Source: PLoS One. 2017 Nov 28;12(11):e0188568. doi: 10.1371/journal.pone.0188568 (PMC5705105; doi:10.1371/journal.pone.0188568)
Supplement: S4 Table — (DOCX) [file pone.0188568.s007.docx]

##### S4 Table Effect of different feeding regimes on the proportion of fused *Acropora* recruits (% fused). T0: experiment commencement, T1: T0 + 46 days, T2: T0 + 93 days.

| **Species** | **Time** | **ATF** | **CTL** | **RAW** | **ROT** |
| --- | --- | --- | --- | --- | --- |
| ***A. hyacinthus*** | **T0** | 38.2 ± 3^a^ | 38.9 ± 3.72^a^ | 35.3 ± 3.73^a^ | 37.7 ± 0.53^a^ |
|  | **T1** | 69.6 ± 3.15^a^ | 66.9 ± 6.45^a^ | 76.6 ± 4.32^a^ | 68.7 ± 3.05^a^ |
|  | **T2** | 85.2 ± 1.43^a^ | 75.3 ± 7.34^a^ | 94.8 ± 2.97^a^ | 72.3 ± 7.29^a^ |
|  | | | | | |
| ***A. loripes*** | **T0** | 32.9 ± 1.84^ab^ | 27.3 ± 2.56^b^ | 33.5 ± 2.54^ab^ | 40.9 ± 1.75^a^ |
|  | **T1** | 54.8 ± 4.36^b^ | 54.3 ± 4.43^b^ | 71.4 ± 1.17^a^ | 69.6 ± 6.53^ab^ |
|  | **T2** | 70.4 ± 3.5^a^ | 66.8 ± 2.54^a^ | 96.4 ± 2.17^b^ | 83.6 ± 2.47^ab^ |
|  | | | | | |
| ***A. millepora*** | **T0** | 34.3 ± 4.96^a^ | 32.7 ± 5^a^ | 37.3 ± 5.12^a^ | 26.3 ± 0.14^a^ |
|  | **T1** | 48.4 ± 9.36^a^ | 51.1 ± 7.1^a^ | 66.2 ± 8.22^a^ | 46.5 ± 3.13^a^ |
|  | **T2** | 55.9 ± 5.96^b^ | 45.7 ± 2.35^b^ | 91.3 ± 4.34^a^ | 56.2 ± 10.1^b^ |
|  | | | | | |
| ***A. tenuis*** | **T0** | 29.7 ± 0.49^a^ | 35 ± 3.43^a^ | 26.5 ± 2.88^a^ | 33.8 ± 0.13^a^ |
|  | **T1** | 41.2 ± 1.63^b^ | 44.3 ± 6.96^b^ | 64.6 ± 1.81^a^ | 52.5 ± 2.57^ab^ |
|  | **T2** | 47.9 ± 6.11^a^ | 40.6 ± 4.06^a^ | 94.1 ± 3.93^b^ | 57.9 ± 1.93^ab^ |

Values are presented as means ± SEM. Values in the same row that do not share the same superscripts are significantly different (*P*<0.05).
