# Supplementary material for: Influence of different feeding regimes on the survival, growth, and biochemical composition of Acropora coral recruits
Source: PLoS One. 2017 Nov 28;12(11):e0188568. doi: 10.1371/journal.pone.0188568 (PMC5705105; doi:10.1371/journal.pone.0188568)
Supplement: S5 Table — (DOCX) [file pone.0188568.s008.docx]

##### S5 Table Effect of different feeding regimes on the total lipid and ash composition of *Acropora* recruits after 93 days.

| **Species** |  | **ATF** | **CTL** | **RAW** | | **ROT** |
| --- | --- | --- | --- | --- | --- | --- |
| ***A. hyacinthus*** | **Lipid** *(mg g sample^-1^)* | 2.85 ± 0.15^a^ | 3.2 ± 0.26^a^ | 3.51 ± 0.09^a^ | | 2.14 ± 0.73^a^ |
|  | **Lipid** *(mg g AFDW^-1^)* | 29.1 ± 0.81^a^ | 34.1 ± 3.4^a^ | 33.5 ± 1.33^a^ | | 21.5 ± 7.38^a^ |
|  | **Ash** *(mg g sample^-1^)* | 90.2 ± 0.38^a^ | 90.5 ± 0.27^a^ | 89.5 ± 0.39^a^ | | 90.1 ± 0^a^ |
|  | | | | |  |  |
| ***A. loripes*** | **Lipid** *(mg g sample^-1^)* | 3.38 ± 0.45^a^ | 2.26 ± 0.47^a^ | 3.61 ± 0.39^a^ | | 3.01 ± 0.09^a^ |
|  | **Lipid** *(mg g AFDW^-1^)* | 32 ± 3.86^a^ | 21.9 ± 8.49^a^ | 36.9 ± 5.48^a^ | | 32.8 ± 0.94^a^ |
|  | **Ash** *(mg g sample^-1^)* | 89.5 ± 0.24^a^ | 91.3 ± 0.39^a^ | 90 ± 0.54^a^ | | 90.8 ± 0^a^ |
|  | | | | |  |  |
| ***A. millepora*** | **Lipid** *(mg g sample^-1^)* | 2.64 ± 0.79^a^ | 4.1 ± 0.22^a^ | 3.5 ± 0.12^a^ | | 3.89 ± 0.44^a^ |
|  | **Lipid** *(mg g AFDW^-1^)* | 23.6 ± 7.07^a^ | 39.3 ± 5.31^a^ | 31.7 ± 1.68^a^ | | 42.9 ± 3.47^a^ |
|  | **Ash** *(mg g sample^-1^)* | 88.8 ± 0^a^ | 89.1 ± 1.3^a^ | 88.9 ± 0.22^a^ | | 91 ± 0.28^a^ |
|  | | | | |  |  |
| ***A. tenuis*** | **Lipid** *(mg g sample^-1^)* | 3.15 ± 0.39^a^ | 2.99 ± 0.26^a^ | 4.71 ± 0.18^a^ | | 3.46 ± 0.12^a^ |
|  | **Lipid** *(mg g AFDW^-1^)* | 27.6 ± 3.07^a^ | 24.2 ± 3.4^a^ | 45.7 ± 0.81^a^ | | 35.2 ± 3.42^a^ |
|  | **Ash** *(mg g sample^-1^)* | 88.6 ± 0.7^a^ | 87.4 ± 0.68^a^ | 89.7 ± 0.28^a^ | | 90 ± 0.64^a^ |

Values are presented as means ± SEM. Values in the same row that do not share the same superscripts are significantly different (*P*<0.05).
