# Supplementary material for: Influence of different feeding regimes on the survival, growth, and biochemical composition of Acropora coral recruits
Source: PLoS One. 2017 Nov 28;12(11):e0188568. doi: 10.1371/journal.pone.0188568 (PMC5705105; doi:10.1371/journal.pone.0188568)
Supplement: S6 Table — (DOCX) [file pone.0188568.s009.docx]

##### S6 Table Fatty acid composition of planktonic larvae for each *Acropora* species (mg g lipid^-1^ and % lipid)

|  | ***A. hyacinthus*** | | ***A. loripes*** | | ***A. millepora*** | | ***A. tenuis*** | | |
| --- | --- | --- | --- | --- | --- | --- | --- | --- | --- |
| *Fatty acids* | *mg g lipid^-1^* | *% fatty acids* | *mg g lipid^-1^* | *% fatty acids* | *mg g lipid^-1^* | *% fatty acids* | *mg g lipid^-1^* | *% fatty acids* |  |
| **10:0** | 15 ± 0.3^a^ | 5.61 ± 0.09_a_ | 12 ± 0.9^b^ | 4.98 ± 0.1_b_ | 14.8 ± 0.1^b^ | 5.51 ± 0.05_b_ | 17.3 ± 0.61^c^ | 8.07 ± 0.23_c_ |  |
| **12:0** | 0.93 ± 0.01^a^ | 0.35 ± 0_a_ | 0.8 ± 0.05^b^ | 0.33 ± 0.01_ab_ | 0.6 ± 0.01^b^ | 0.22 ± 0_b_ | 0.69 ± 0.03^c^ | 0.32 ± 0.01_c_ |  |
| **14:0** | 20.5 ± 0.06^a^ | 7.64 ± 0.06_a_ | 15.8 ± 0.92^b^ | 6.54 ± 0.04_b_ | 18.3 ± 0.21^c^ | 6.78 ± 0.05_c_ | 24 ± 1.29^d^ | 11.1 ± 0.09_d_ |  |
| **16:0** | 110 ± 0.46^a^ | 41.2 ± 0.32_a_ | 109 ± 5.79^ab^ | 45.1 ± 0.22_b_ | 99.1 ± 1.08^bc^ | 36.8 ± 0.28_c_ | 81 ± 4.47^c^ | 37.6 ± 0.27_c_ |  |
| **18:0** | 8.74 ± 0.74^a^ | 3.26 ± 0.26_a_ | 8.49 ± 0.22^b^ | 3.55 ± 0.18_b_ | 7.83 ± 0.41^b^ | 2.9 ± 0.11_bc_ | 12.8 ± 0.95^b^ | 5.95 ± 0.09_c_ |  |
| **∑SFA** | 160 ± 1.14^a^ | 59.6 ± 0.33_a_ | 149 ± 8.06^ab^ | 62 ± 0.3_b_ | 145 ± 1.74^ab^ | 53.8 ± 0.42_c_ | 140 ± 7.43^b^ | 64.9 ± 0.54_d_ |  |
| **16:1n-7** | 17.4 ± 0.26^a^ | 6.49 ± 0.06_a_ | 12.8 ± 0.65^b^ | 5.32 ± 0.04_b_ | 22.4 ± 0.23^c^ | 8.34 ± 0.05_c_ | 9.79 ± 0.46^d^ | 4.56 ± 0.07_d_ |  |
| **18:1n-9** | 20.4 ± 0.2^a^ | 7.6 ± 0.06_a_ | 23.2 ± 1.24^a^ | 9.64 ± 0.04_a_ | 25.9 ± 0.36^b^ | 9.6 ± 0.05_b_ | 14.3 ± 0.86^c^ | 6.64 ± 0.04_b_ |  |
| **20:1n-11** | 2.7 ± 0.04^a^ | 1.01 ± 0.01_a_ | 2.65 ± 0.18^a^ | 1.1 ± 0.02_ab_ | 2.77 ± 0.05^a^ | 1.03 ± 0.01_b_ | 1.41 ± 0.09^b^ | 0.65 ± 0.01_c_ |  |
| **∑MUFA** | 48.7 ± 0.65^a^ | 18.2 ± 0.18_a_ | 45.6 ± 2.44^b^ | 18.9 ± 0.08_b_ | 59 ± 0.97^b^ | 21.9 ± 0.14_c_ | 34.4 ± 2.06^c^ | 16 ± 0.1_d_ |  |
| **18:3n-6** | 7.49 ± 0.04^a^ | 2.79 ± 0.02_a_ | 6.06 ± 0.45^b^ | 2.51 ± 0.06_b_ | 10.4 ± 0.66^c^ | 3.87 ± 0.21_c_ | 2.47 ± 0.32^d^ | 1.14 ± 0.07_d_ |  |
| **20:4n-6** | 0.1 ± 0^a^ | 0.04 ± 0_a_ | 0.1 ± 0.01^ab^ | 0.04 ± 0_b_ | 0.11 ± 0^b^ | 0.04 ± 0_bc_ | 0.37 ± 0.05^b^ | 0.17 ± 0.02_c_ |  |
| **20:5n-3** | 24.5 ± 0.17^a^ | 9.13 ± 0.1_a_ | 22.5 ± 1.37^b^ | 9.33 ± 0.06_b_ | 26.2 ± 0.35^b^ | 9.72 ± 0.08_b_ | 15.6 ± 0.99^c^ | 7.26 ± 0.08_c_ |  |
| **22:6n-3** | 5.46 ± 0.06^a^ | 2.04 ± 0.03_a_ | 4.98 ± 0.43^a^ | 2.06 ± 0.08_ab_ | 4.28 ± 0.6^a^ | 1.59 ± 0.21_ab_ | 4.55 ± 0.82^a^ | 2.07 ± 0.25_b_ |  |
| **∑PUFA** | 59.7 ± 0.5^a^ | 22.2 ± 0.16_a_ | 46.1 ± 3.19^b^ | 19.1 ± 0.29_a_ | 65.4 ± 2.18^c^ | 24.3 ± 0.53_b_ | 41.3 ± 3.82^c^ | 19.1 ± 0.56_b_ |  |
| **TOTAL** | 346 ± 2.13^a^ | 100 ± 0_a_ | 314 ± 17.8^a^ | 100 ± 0_a_ | 355 ± 6.58^ab^ | 100 ± 0_a_ | 274 ± 17.5^b^ | 100 ± 0_a_ |  |
| **∑n-3 PUFA** | 12.3 ± 0.04^a^ | 4.58 ± 0.02_a_ | 10.3 ± 0.74^b^ | 4.27 ± 0.08_b_ | 17.1 ± 0.92^c^ | 6.34 ± 0.29_c_ | 5.54 ± 0.67^d^ | 2.55 ± 0.15_d_ |  |
| **∑n-6 PUFA** | 12.2 ± 0.04^a^ | 4.54 ± 0.02_a_ | 10.2 ± 0.73^b^ | 4.24 ± 0.08_b_ | 17 ± 0.93^c^ | 6.29 ± 0.3_c_ | 5.4 ± 0.66^d^ | 2.48 ± 0.15_d_ |  |
| **∑n-3 LC PUFA** | 39 ± 0.2^a^ | 14.5 ± 0.15_a_ | 35.6 ± 2.33^ab^ | 14.8 ± 0.19_ab_ | 39.1 ± 1.11^ab^ | 14.5 ± 0.25_ab_ | 29.2 ± 2.48^b^ | 13.5 ± 0.3_b_ |  |
| **∑n-6 LC PUFA** | 9.19 ± 0.12^a^ | 3.43 ± 0.06_a_ | 8.35 ± 0.69^a^ | 3.45 ± 0.12_a_ | 7.75 ± 0.93^a^ | 2.87 ± 0.32_a_ | 8.45 ± 1.21^a^ | 3.87 ± 0.32_a_ |  |
| **n-3:n-6** | 0.32 ± 0^a^ | 0.32 ± 0_a_ | 0.29 ± 0^b^ | 0.29 ± 0_b_ | 0.44 ± 0.01^c^ | 0.44 ± 0.01_c_ | 0.19 ± 0.01^d^ | 0.19 ± 0.01_d_ |  |
| **LC n-3:LC n-6** | 1.33 ± 0.02^a^ | 1.33 ± 0.02_a_ | 1.23 ± 0.02^b^ | 1.23 ± 0.02_b_ | 2.24 ± 0.17^c^ | 2.24 ± 0.17_c_ | 0.64 ± 0.01^d^ | 0.64 ± 0.01_d_ |  |
| **EPA:DHA** | 76.6 ± 2.18^a^ | 76.6 ± 2.18_a_ | 63.5 ± 2.76^b^ | 63.5 ± 2.76_b_ | 95.8 ± 4.49^c^ | 95.8 ± 4.49_c_ | 6.74 ± 0.53^d^ | 6.74 ± 0.53_d_ |  |
| **EPA:ARA** | 1.37 ± 0.02^a^ | 1.37 ± 0.02_a_ | 1.22 ± 0.02^b^ | 1.22 ± 0.02_b_ | 2.53 ± 0.24^c^ | 2.53 ± 0.24_c_ | 0.56 ± 0.03^d^ | 0.56 ± 0.03_d_ |  |

Values are presented as means ± SEM. Values in the same row that do not share a superscript are significantly different (*P*<0.05). Values in the same row that do not share a subscript are significantly different (*P*<0.05).
