# Supplementary material for: Influence of different feeding regimes on the survival, growth, and biochemical composition of Acropora coral recruits
Source: PLoS One. 2017 Nov 28;12(11):e0188568. doi: 10.1371/journal.pone.0188568 (PMC5705105; doi:10.1371/journal.pone.0188568)
Supplement: S7 Table — (DOCX) [file pone.0188568.s010.docx]

##### S7 Table Effect of different feeding regimes on the fatty acid composition of *Acropora hyacinthus* recruits after 93 days (mg g lipid^-1^ and % lipid)

| ***A. hyacinthus*** | | | | | | | | | |
| --- | --- | --- | --- | --- | --- | --- | --- | --- | --- |
|  | **ATF** | | **CTL** | | **RAW** | | **ROT** | | |
| *Fatty acids* | *mg g lipid^-1^* | *% fatty acids* | *mg g lipid^-1^* | *% fatty acids* | *mg g lipid^-1^* | *% fatty acids* | *mg g lipid^-1^* | *% fatty acids* |  |
| **10:0** | 0.55 ± 0.07^a^ | 0.24 ± 0.04_a_ | 0.67 ± 0.39^a^ | 0.28 ± 0.15_a_ | 0.65 ± 0.23^a^ | 0.33 ± 0.12_a_ | 0.46 ± 0.03^a^ | 0.21 ± 0.01_a_ |  |
| **12:0** | 5.92 ± 0.49^a^ | 2.61 ± 0.27_a_ | 4.95 ± 0.85^a^ | 2.3 ± 0.31_a_ | 4.97 ± 0.75^a^ | 2.51 ± 0.45_a_ | 4.08 ± 0^a^ | 1.92 ± 0.24_a_ |  |
| **14:0** | 12.8 ± 1.54^a^ | 5.52 ± 0.5_a_ | 11.7 ± 2.5^a^ | 5.35 ± 0.67_a_ | 10.1 ± 1.03^a^ | 5.01 ± 0.38_a_ | 11.7 ± 2.18^a^ | 5.21 ± 0.35_a_ |  |
| **16:0** | 64.7 ± 3.25^a^ | 28.2 ± 0.8_a_ | 63.2 ± 6.84^a^ | 29.7 ± 0.6_a_ | 58.8 ± 3.28^a^ | 29.2 ± 0.84_a_ | 62.1 ± 10.7^a^ | 27.8 ± 1.43_a_ |  |
| **18:0** | 17.7 ± 0.63^a^ | 7.72 ± 0.04_ab_ | 17.2 ± 1.52^a^ | 8.19 ± 0.53_a_ | 13.3 ± 0.53^a^ | 6.62 ± 0.29_b_ | 16.3 ± 1.6^a^ | 7.47 ± 0.2_ab_ |  |
| **∑SFA** | 110 ± 4.22^a^ | 47.8 ± 0.53_a_ | 105 ± 11.4^a^ | 49.4 ± 1.02_a_ | 94.3 ± 4.82^a^ | 46.9 ± 1.86_a_ | 102 ± 14.2^a^ | 45.9 ± 0.79_a_ |  |
| **16:1n-7** | 10.8 ± 0.76^a^ | 4.74 ± 0.42^a^ | 9.93 ± 1.09^ab^ | 4.71 ± 0.33^a^ | 7.66 ± 0.71^b^ | 3.78 ± 0.21^a^ | 11.1 ± 1.53^ab^ | 5.02 ± 0.08^a^ |  |
| **18:1n-9** | 9.98 ± 1.1^a^ | 4.33 ± 0.38_a_ | 10.2 ± 3.11^a^ | 4.55 ± 1.01_a_ | 6.97 ± 0.43^a^ | 3.47 ± 0.23_a_ | 8.45 ± 1.44^a^ | 3.78 ± 0.19_a_ |  |
| **20:1n-11** | 17.8 ± 0.73^ab^ | 7.83 ± 0.54_a_ | 15 ± 1.5^b^ | 7.49 ± 1.43_a_ | 20.1 ± 1.87^ab^ | 9.98 ± 0.92_a_ | 20.3 ± 0.65^a^ | 9.49 ± 0.88_a_ |  |
| **∑MUFA** | 58.7 ± 1.76^a^ | 25.7 ± 1.16_a_ | 52.3 ± 4.88^a^ | 24.6 ± 0.24_a_ | 46.4 ± 3.04^a^ | 23 ± 0.9_a_ | 54.1 ± 5.56^a^ | 24.7 ± 0.52_a_ |  |
| **18:3n-6** | 13.9 ± 3.03^a^ | 6.01 ± 1.21_a_ | 13.4 ± 2.44^a^ | 6.17 ± 0.59_a_ | 16.2 ± 0.36^a^ | 8.1 ± 0.44_a_ | 12.6 ± 0.63^a^ | 5.84 ± 0.43_a_ |  |
| **20:4n-6** | 12.3 ± 0.95^a^ | 5.39 ± 0.41_a_ | 11.3 ± 0.6^a^ | 5.43 ± 0.31_a_ | 10.1 ± 0.59^a^ | 5.04 ± 0.25_a_ | 13.6 ± 1.52^a^ | 6.18 ± 0.07_a_ |  |
| **20:5n-3** | 10.5 ± 0.42^a^ | 4.6 ± 0.28_a_ | 9.42 ± 0.68^a^ | 4.68 ± 0.77_a_ | 11.8 ± 1.18^a^ | 5.84 ± 0.53_a_ | 12.6 ± 0.83^a^ | 5.82 ± 0.34_a_ |  |
| **22:6n-3** | 8.47 ± 0.41^ab^ | 3.7 ± 0.11_b_ | 7.12 ± 0.45^b^ | 3.43 ± 0.3_b_ | 9.87 ± 0.86^a^ | 4.9 ± 0.35_a_ | 9.28 ± 1.14^ab^ | 4.22 ± 0_a_ |  |
| **∑PUFA** | 60.6 ± 4.56^a^ | 26.4 ± 1.41_b_ | 54.5 ± 3.72^a^ | 26 ± 1.23_ab_ | 60.2 ± 2.44^a^ | 30 ± 1.14_a_ | 64.6 ± 7.39^a^ | 29.4 ± 0.27_a_ |  |
| **TOTAL** | 229 ± 7.61^a^ | 100 ± 0_a_ | 212 ± 19.6^a^ | 100 ± 0_a_ | 201 ± 7.18^a^ | 100 ± 0_a_ | 220 ± 27.1^a^ | 100 ± 0_a_ |  |
| **∑n-3 PUFA** | 24.2 ± 0.54^a^ | 10.6 ± 0.41_a_ | 20.9 ± 0.42^a^ | 10.2 ± 1.13_a_ | 25.8 ± 2.15^a^ | 12.8 ± 0.86_a_ | 27.3 ± 3.11^a^ | 12.4 ± 0.12_a_ |  |
| **∑n-6 PUFA** | 23.2 ± 0.48^ab^ | 10.1 ± 0.34_a_ | 20 ± 0.39^b^ | 9.77 ± 1.12_a_ | 25.2 ± 2.29^ab^ | 12.5 ± 0.93_a_ | 26.4 ± 2.75^a^ | 12 ± 0.23_a_ |  |
| **∑n-3 LC PUFA** | 34.4 ± 4.87^a^ | 14.9 ± 1.81_a_ | 31.6 ± 4.24^a^ | 14.8 ± 0.84_a_ | 32.7 ± 0.35^a^ | 16.3 ± 0.62_a_ | 35.8 ± 4.53^a^ | 16.2 ± 0.05_a_ |  |
| **∑n-6 LC PUFA** | 17.9 ± 1.17^a^ | 7.82 ± 0.48_a_ | 15.9 ± 0.78^a^ | 7.63 ± 0.41_a_ | 14.8 ± 0.9^a^ | 7.33 ± 0.34_a_ | 19.7 ± 2.2^a^ | 9 ± 0.11_a_ |  |
| **n-3:n-6** | 0.77 ± 0.14^a^ | 0.77 ± 0.14_a_ | 0.7 ± 0.09^a^ | 0.7 ± 0.09_a_ | 0.79 ± 0.06^a^ | 0.79 ± 0.06_a_ | 0.76 ± 0.01^a^ | 0.76 ± 0.01_a_ |  |
| **LC n-3:LC n-6** | 1.32 ± 0.12^b^ | 1.32 ± 0.12_b_ | 1.27 ± 0.08^b^ | 1.27 ± 0.08_b_ | 1.7 ± 0.06^a^ | 1.7 ± 0.06_a_ | 1.34 ± 0.01^ab^ | 1.34 ± 0.01_ab_ |  |
| **EPA:DHA** | 1.26 ± 0.11^a^ | 1.26 ± 0.11_a_ | 1.35 ± 0.16^a^ | 1.35 ± 0.16_a_ | 1.19 ± 0.02^a^ | 1.19 ± 0.02_a_ | 1.38 ± 0.08^a^ | 1.38 ± 0.08_a_ |  |
| **EPA:ARA** | 0.88 ± 0.11^a^ | 0.88 ± 0.11_a_ | 0.85 ± 0.1^a^ | 0.85 ± 0.1_a_ | 1.15 ± 0.05^a^ | 1.15 ± 0.05_a_ | 0.94 ± 0.04^a^ | 0.94 ± 0.04_a_ |  |

Values are presented as means ± SEM. Values in the same row that do not share a superscript are significantly different (*P*<0.05). Values in the same row that do not share a subscript are significantly different (*P*<0.05).
