# Supplementary material for: Influence of different feeding regimes on the survival, growth, and biochemical composition of Acropora coral recruits
Source: PLoS One. 2017 Nov 28;12(11):e0188568. doi: 10.1371/journal.pone.0188568 (PMC5705105; doi:10.1371/journal.pone.0188568)
Supplement: S8 Table — (DOCX) [file pone.0188568.s011.docx]

##### S8 Table Effect of different feeding regimes on the fatty acid composition of *Acropora loripes* recruits after 93 days (mg g lipid^-1^ and % lipid)

| ***A. loripes*** | | | | | | | | | |
| --- | --- | --- | --- | --- | --- | --- | --- | --- | --- |
|  | **ATF** | | **CTL** | | **RAW** | | **ROT** | | |
| *Fatty acids* | *mg g lipid^-1^* | *% fatty acids* | *mg g lipid^-1^* | *% fatty acids* | *mg g lipid^-1^* | *% fatty acids* | *mg g lipid^-1^* | *% fatty acids* |  |
| **10:0** | 0.88 ± 0.28^a^ | 0.4 ± 0.09_a_ | 1.32 ± 0.55^a^ | 0.56 ± 0.2_a_ | 0.8 ± 0.07^a^ | 0.44 ± 0.02_a_ | 1.28 ± 0.15^a^a | 0.66 ± 0.08_a_a |  |
| **12:0** | 9.24 ± 1.57^a^ | 4.51 ± 0.44_ab_ | 7.32 ± 1.6^ab^ | 3.25 ± 0.54_bc_ | 5 ± 0.46^b^ | 2.79 ± 0.24_c_ | 10.6 ± 1.27^a^ | 5.43 ± 0.71_a_ |  |
| **14:0** | 12.5 ± 1.13^a^ | 6.23 ± 0.49_a_ | 14.1 ± 2.65^a^ | 6.24 ± 0.71_a_ | 9.51 ± 1.12^a^ | 5.27 ± 0.44_a_ | 11.6 ± 0.4^a^ | 5.96 ± 0.14_a_ |  |
| **16:0** | 65.6 ± 6.52^a^ | 32.3 ± 1.04_a_ | 67.9 ± 5.2^a^ | 30.9 ± 1.41_a_ | 51.4 ± 3.47^a^ | 28.6 ± 1.08_a_ | 58.3 ± 0.36^a^ | 29.9 ± 0.49_a_ |  |
| **18:0** | 22.6 ± 1.35^a^ | 11.6 ± 1.66_a_ | 19.8 ± 1.1^a^ | 9.07 ± 0.45_a_ | 15.6 ± 1.21^b^ | 8.68 ± 0.34_a_ | 19.3 ± 0.54^ab^ | 9.91 ± 0.38_a_ |  |
| **∑SFA** | 118 ± 9.6^a^ | 58.7 ± 3.55_a_ | 117 ± 10.4^a^ | 53.1 ± 1.4_ab_ | 89.2 ± 6.1^a^ | 49.6 ± 1.68_b_ | 107 ± 2.04^a^ | 54.9 ± 1.6_ab_ |  |
| **16:1n-7** | 6.92 ± 2.63^a^ | 2.95 ± 1.1^a^ | 9.54 ± 0.77^a^ | 4.33 ± 0.07^a^ | 6.73 ± 0.91^a^ | 3.67 ± 0.21^a^ | 8.37 ± 0.55^a^ | 4.28 ± 0.24^a^ |  |
| **18:1n-9** | 9.57 ± 1.04^a^ | 4.7 ± 0.19_a_ | 11.5 ± 2.36^a^ | 5.05 ± 0.67_a_ | 6.78 ± 0.76^a^ | 3.73 ± 0.18_b_ | 7.98 ± 0.6^a^ | 4.08 ± 0.27_ab_ |  |
| **20:1n-11** | 11.3 ± 2^a^ | 5.36 ± 0.37_b_ | 11.9 ± 1.7^a^ | 5.64 ± 1.1_b_ | 17.6 ± 2.23^a^ | 9.57 ± 0.54_a_ | 12.6 ± 1.23^a^ | 6.44 ± 0.57_ab_ |  |
| **∑MUFA** | 41.4 ± 9^a^ | 19.2 ± 2.39_a_ | 46.2 ± 2.8^a^ | 21 ± 0.34_a_ | 37.2 ± 4.21^a^ | 20.3 ± 0.55_a_ | 40.6 ± 0.05^a^ | 20.8 ± 0.24_a_ |  |
| **18:3n-6** | 10.9 ± 2.08^a^ | 5.44 ± 0.9_a_ | 13.8 ± 2.13^a^ | 6.23 ± 0.76_a_ | 13.4 ± 0.75^a^ | 7.47 ± 0.34_a_ | 11.3 ± 1.94^a^ | 5.75 ± 0.94_a_ |  |
| **20:4n-6** | 9.05 ± 1.38^a^ | 4.34 ± 0.22_a_ | 11.3 ± 0.6^a^ | 5.18 ± 0.14_a_ | 9.72 ± 1.43^a^ | 5.25 ± 0.35_a_ | 8.94 ± 0.67^a^ | 4.57 ± 0.3_a_ |  |
| **20:5n-3** | 7.06 ± 1.64^a^ | 3.26 ± 0.45_b_ | 8.85 ± 0.44^a^ | 4.13 ± 0.46_ab_ | 11.2 ± 1.92^a^ | 6.02 ± 0.6_a_ | 8.52 ± 0.51^a^ | 4.36 ± 0.22_ab_ |  |
| **22:6n-3** | 5.8 ± 1.23^a^ | 2.71 ± 0.36_b_ | 6.9 ± 0.25^a^ | 3.17 ± 0.14_b_ | 7.91 ± 1.01^a^ | 4.3 ± 0.2_a_ | 6.02 ± 0.71^a^ | 3.07 ± 0.33_b_ |  |
| **∑PUFA** | 45.8 ± 7.14^a^ | 22 ± 1.45_b_ | 56.4 ± 2.79^a^ | 25.8 ± 1.07_ab_ | 55.2 ± 6.66^a^ | 30.1 ± 1.13_a_ | 47.5 ± 4.08^a^ | 24.3 ± 1.84_ab_ |  |
| **TOTAL** | 205 ± 24.8^a^ | 100 ± 0_a_ | 220 ± 15.3^a^ | 100 ± 0_a_ | 182 ± 16.7^a^ | 100 ± 0_a_ | 195 ± 1.99^a^ | 100 ± 0_a_ |  |
| **∑n-3 PUFA** | 17.5 ± 3.66^a^ | 8.18 ± 0.88_b_ | 20.3 ± 0.33^a^ | 9.4 ± 0.7_ab_ | 22.8 ± 3.36^a^ | 12.3 ± 0.87_a_ | 18.6 ± 1.03^a^ | 9.5 ± 0.43_ab_ |  |
| **∑n-6 PUFA** | 16.6 ± 3.63^a^ | 7.74 ± 0.93_b_ | 19.5 ± 0.26^a^ | 9.01 ± 0.68_ab_ | 22.4 ± 3.37^a^ | 12.1 ± 0.9_a_ | 17.9 ± 1.18^a^ | 9.16 ± 0.51_ab_ |  |
| **∑n-3 LC PUFA** | 27.1 ± 3.89^a^ | 13.3 ± 1.25_b_ | 34.8 ± 2.95^a^ | 15.8 ± 0.8_ab_ | 30.9 ± 3.07^a^ | 17 ± 0.22_a_ | 27.7 ± 2.85^a^ | 14.2 ± 1.31_ab_ |  |
| **∑n-6 LC PUFA** | 13.4 ± 2.19^a^ | 6.4 ± 0.39_b_ | 16.9 ± 0.8^a^ | 7.71 ± 0.18_a_ | 14.6 ± 1.96^a^ | 7.9 ± 0.4_a_ | 13.2 ± 0.89^a^ | 6.74 ± 0.38_ab_ |  |
| **n-3:n-6** | 0.64 ± 0.11^a^ | 0.64 ± 0.11_a_ | 0.6 ± 0.06^a^ | 0.6 ± 0.06_ab_ | 0.72 ± 0.04^a^ | 0.72 ± 0.04_b_ | 0.68 ± 0.03^a^ | 0.68 ± 0.03_a_ |  |
| **LC n-3:LC n-6** | 1.2 ± 0.09^b^ | 1.2 ± 0.09_b_ | 1.16 ± 0.07^b^ | 1.16 ± 0.07_b_ | 1.52 ± 0.05^a^ | 1.52 ± 0.05_b_ | 1.36 ± 0^ab^ | 1.36 ± 0_a_ |  |
| **EPA:DHA** | 1.21 ± 0.09^a^ | 1.21 ± 0.09_a_ | 1.29 ± 0.1^a^ | 1.29 ± 0.1_a_ | 1.39 ± 0.08^a^ | 1.39 ± 0.08_a_ | 1.44 ± 0.09^a^ | 1.44 ± 0.09_a_ |  |
| **EPA:ARA** | 0.74 ± 0.08^b^ | 0.74 ± 0.08_b_ | 0.79 ± 0.07^b^ | 0.79 ± 0.07_b_ | 1.14 ± 0.05^a^ | 1.14 ± 0.05_a_ | 0.96 ± 0.01^ab^ | 0.96 ± 0.01_ab_ |  |

Values are presented as means ± SEM. Values in the same row that do not share a superscript are significantly different (*P*<0.05). Values in the same row that do not share a subscript are significantly different (*P*<0.05).
