# Supplementary material for: Influence of different feeding regimes on the survival, growth, and biochemical composition of Acropora coral recruits
Source: PLoS One. 2017 Nov 28;12(11):e0188568. doi: 10.1371/journal.pone.0188568 (PMC5705105; doi:10.1371/journal.pone.0188568)
Supplement: S9 Table — (DOCX) [file pone.0188568.s012.docx]

##### S9 Table Effect of different feeding regimes on the fatty acid composition of *Acropora millepora* recruits after 93 days (mg g lipid^-1^ and % lipid)

| ***A. millepora*** | | | | | | | | | |
| --- | --- | --- | --- | --- | --- | --- | --- | --- | --- |
|  | **ATF** | | **CTL** | | **RAW** | | **ROT** | | |
| *Fatty acids* | *mg g lipid^-1^* | *% fatty acids* | *mg g lipid^-1^* | *% fatty acids* | *mg g lipid^-1^* | *% fatty acids* | *mg g lipid^-1^* | *% fatty acids* |  |
| **10:0** | 0.53 ± 0.1^ab^ | 0.18 ± 0.04_b_ | 0.15 ± 0.12^b^ | 0.06 ± 0.05_c_ | 0.96 ± 0.21^a^ | 0.43 ± 0.09_a_ | 0.32 ± 0.01^b^ | 0.19 ± 0.02_bc_ |  |
| **12:0** | 6.75 ± 0.98^a^ | 2.29 ± 0.55_a_ | 3.59 ± 0.79^b^ | 2.05 ± 0.25_a_ | 4.97 ± 0.96^ab^ | 2.24 ± 0.4_a_ | 3.51 ± 0.07^ab^ | 2.11 ± 0.27_a_ |  |
| **14:0** | 17 ± 1.62^a^ | 5.4 ± 0.25_a_ | 10.3 ± 2.87^b^ | 5.7 ± 0.87_a_ | 10.7 ± 0.78^ab^ | 4.86 ± 0.4_a_ | 8.05 ± 1^b^ | 4.67 ± 0.08_a_ |  |
| **16:0** | 101 ± 5.81^a^ | 32.3 ± 1.48_ab_ | 61.6 ± 8.07^b^ | 36 ± 2.4_a_ | 63.3 ± 2.01^b^ | 28.7 ± 0.6_b_ | 53.9 ± 7.76^b^ | 31.1 ± 1.19_ab_ |  |
| **18:0** | 30 ± 1.22^a^ | 9.64 ± 0.62_a_ | 16.2 ± 1.1^b^ | 9.73 ± 0.96_a_ | 16.2 ± 1.35^b^ | 7.33 ± 0.44_b_ | 14.1 ± 0.96^b^ | 8.29 ± 0.33_ab_ |  |
| **∑SFA** | 168 ± 7.53^a^ | 54 ± 3.38_ab_ | 101 ± 12.7^b^ | 59.2 ± 3.46_a_ | 106 ± 6.47^b^ | 48.1 ± 2.15_b_ | 86.3 ± 9.49^b^ | 50.2 ± 0.14_ab_ |  |
| **16:1n-7** | 11.7 ± 4.4^a^ | 3.34 ± 1.25^a^ | 4.11 ± 3.05^a^ | 1.75 ± 1.26^a^ | 6.34 ± 2.29^a^ | 2.94 ± 1.07^a^ | 5.77 ± 3.51^a^ | 2.9 ± 1.73^a^ |  |
| **18:1n-9** | 18.1 ± 3.41^a^ | 5.63 ± 0.69_a_ | 9.27 ± 2.94^b^ | 4.95 ± 0.82_ab_ | 7.92 ± 0.82^ab^ | 3.58 ± 0.31_b_ | 6.32 ± 0.78^b^ | 3.66 ± 0.06_ab_ |  |
| **20:1n-11** | 21.9 ± 2.53^ab^ | 6.85 ± 0.22_a_ | 10.5 ± 3.23^b^ | 6.19 ± 1.91_a_ | 22.2 ± 2.09^a^ | 10.1 ± 0.87_a_ | 16.3 ± 0.34^ab^ | 9.81 ± 1.25_a_ |  |
| **∑MUFA** | 71.1 ± 10^a^ | 22 ± 1.2_a_ | 32.6 ± 7.09^b^ | 18 ± 1.38_a_ | 47.4 ± 1.72^ab^ | 21.5 ± 0.88_a_ | 40.7 ± 7.32^ab^ | 23.2 ± 1.77_a_ |  |
| **18:3n-6** | 16.5 ± 2.8^ab^ | 5.26 ± 0.75_b_ | 9.88 ± 2.89^ab^ | 5.27 ± 0.74_ab_ | 16.6 ± 1.27^a^ | 7.47 ± 0.39_a_ | 8.69 ± 1.44^b^ | 5.44 ± 1.42_ab_ |  |
| **20:4n-6** | 11.8 ± 4.59^a^ | 3.33 ± 1.3_b_ | 8.34 ± 1.69^a^ | 4.64 ± 0.44_ab_ | 12.3 ± 0.38^a^ | 5.59 ± 0.25_a_ | 8.46 ± 0.23^a^ | 5.04 ± 0.41_ab_ |  |
| **20:5n-3** | 11.8 ± 2.28^ab^ | 3.64 ± 0.5_b_ | 6.99 ± 1.47^b^ | 4.06 ± 0.79_ab_ | 13.8 ± 1.71^a^ | 6.28 ± 0.77_a_ | 9.43 ± 0.32^ab^ | 5.6 ± 0.42_ab_ |  |
| **22:6n-3** | 12.2 ± 3.76^ab^ | 3.58 ± 0.87_ab_ | 4.44 ± 0.97^b^ | 2.47 ± 0.36_b_ | 9.95 ± 0.97^a^ | 4.52 ± 0.45_a_ | 5.98 ± 0.38^ab^ | 3.53 ± 0.15_ab_ |  |
| **∑PUFA** | 78.3 ± 13.7^a^ | 24 ± 2.21_ab_ | 40.8 ± 8.45^a^ | 22.7 ± 2.24_b_ | 66.9 ± 3.17^a^ | 30.4 ± 1.31_a_ | 44.6 ± 1.58^a^ | 26.5 ± 1.92_ab_ |  |
| **TOTAL** | 318 ± 31.1^a^ | 100 ± 0_a_ | 175 ± 27.6^b^ | 100 ± 0_a_ | 221 ± 5.97^b^ | 100 ± 0_a_ | 172 ± 18.4^b^ | 100 ± 0_a_ |  |
| **∑n-3 PUFA** | 32.5 ± 5.77^a^ | 9.94 ± 1_a_ | 14.9 ± 2.9^a^ | 8.43 ± 1.21_a_ | 28 ± 2.53^a^ | 12.7 ± 1.14_a_ | 19.6 ± 1.54^a^ | 11.5 ± 0.33_a_ |  |
| **∑n-6 PUFA** | 30.2 ± 5.69^a^ | 9.2 ± 1.04_a_ | 14 ± 2.75^a^ | 7.98 ± 1.25_a_ | 27.5 ± 2.63^a^ | 12.5 ± 1.18_a_ | 18.9 ± 1.26^a^ | 11.1 ± 0.46_a_ |  |
| **∑n-3 LC PUFA** | 43.4 ± 7.63^a^ | 13.3 ± 1.19_b_ | 25.2 ± 5.9^ab^ | 13.8 ± 1.32_b_ | 37.1 ± 0.8^ab^ | 16.8 ± 0.1_a_ | 23.6 ± 0.09^b^ | 14.2 ± 1.57_ab_ |  |
| **∑n-6 LC PUFA** | 19 ± 5.01^ab^ | 5.61 ± 1.2_a_ | 11.5 ± 2.34^ab^ | 6.39 ± 0.6_a_ | 17.4 ± 0.75^a^ | 7.91 ± 0.43_a_ | 12.7 ± 0.32^ab^ | 7.55 ± 0.62_a_ |  |
| **n-3:n-6** | 0.75 ± 0.04^a^ | 0.75 ± 0.04_a_ | 0.62 ± 0.07^a^ | 0.62 ± 0.07_a_ | 0.76 ± 0.07^a^ | 0.76 ± 0.07_a_ | 0.83 ± 0.07^a^ | 0.83 ± 0.07_a_ |  |
| **LC n-3:LC n-6** | 1.84 ± 0.29^a^ | 1.84 ± 0.29_a_ | 1.24 ± 0.11^a^ | 1.24 ± 0.11_a_ | 1.57 ± 0.08^a^ | 1.57 ± 0.08_a_ | 1.48 ± 0.06^a^ | 1.48 ± 0.06_a_ |  |
| **EPA:DHA** | 1.2 ± 0.23^a^ | 1.2 ± 0.23_a_ | 1.65 ± 0.17^a^ | 1.65 ± 0.17_a_ | 1.38 ± 0.04^a^ | 1.38 ± 0.04_a_ | 1.58 ± 0.05^a^ | 1.58 ± 0.05_a_ |  |
| **EPA:ARA** | 1.09 ± 0.69^a^ | 1.09 ± 0.69_a_ | 0.86 ± 0.11^a^ | 0.86 ± 0.11_a_ | 1.11 ± 0.1^a^ | 1.11 ± 0.1_a_ | 1.11 ± 0.01^a^ | 1.11 ± 0.01_a_ |  |

Values are presented as means ± SEM. Values in the same row that do not share a superscript are significantly different (*P*<0.05). Values in the same row that do not share a subscript are significantly different (*P*<0.05).
