# Supplementary material for: Influence of different feeding regimes on the survival, growth, and biochemical composition of Acropora coral recruits
Source: PLoS One. 2017 Nov 28;12(11):e0188568. doi: 10.1371/journal.pone.0188568 (PMC5705105; doi:10.1371/journal.pone.0188568)
Supplement: S10 Table — (DOCX) [file pone.0188568.s013.docx]

##### S10 Table Effect of different feeding regimes on the fatty acid composition of *Acropora tenuis* recruits after 93 days (mg g lipid^-1^ and % lipid)

| ***A. tenuis*** | | | | | | | | | |
| --- | --- | --- | --- | --- | --- | --- | --- | --- | --- |
|  | **ATF** | | **CTL** | | **RAW** | | **ROT** | | |
| *Fatty acids* | *mg g lipid^-1^* | *% fatty acids* | *mg g lipid^-1^* | *% fatty acids* | *mg g lipid^-1^* | *% fatty acids* | *mg g lipid^-1^* | *% fatty acids* |  |
| **10:0** | 1.11 ± 0.53^b^ | 0.73 ± 0.44_a_ | 3.49 ± 0.6^a^ | 1.29 ± 0.04_a_ | 4.97 ± 1.19^a^ | 2.58 ± 0.57_a_ | 2.67 ± 0.26^ab^ | 1.44 ± 0.12_a_ |  |
| **12:0** | 2.24 ± 0.46^a^ | 1.43 ± 0.42_a_ | 2.9 ± 0.95^a^ | 0.98 ± 0.15_a_ | 3.35 ± 0.42^a^ | 1.75 ± 0.18_a_ | 1.95 ± 0.33^a^ | 1.05 ± 0.16_a_ |  |
| **14:0** | 9.58 ± 2.29^a^ | 4.79 ± 0.29_a_ | 13.2 ± 5.03^a^ | 4.3 ± 0.96_a_ | 7.33 ± 0.81^a^ | 3.84 ± 0.39_a_ | 7.45 ± 0.74^a^ | 4.02 ± 0.36_a_ |  |
| **16:0** | 75.9 ± 11.5^a^ | 40.3 ± 3.05_a_ | 89.3 ± 23.5^a^ | 31.4 ± 2.17_ab_ | 58 ± 3.2^a^ | 30.5 ± 1.83_b_ | 61.4 ± 5.78^a^ | 33.1 ± 2.77_ab_ |  |
| **18:0** | 30.3 ± 9.16^a^ | 14.7 ± 1.46_a_ | 24.8 ± 3.97^a^ | 9.25 ± 0.43_b_ | 18.2 ± 1.37^a^ | 9.59 ± 0.85_b_ | 19.2 ± 0.19^a^ | 10.4 ± 0.21_ab_ |  |
| **∑SFA** | 136 ± 22.7^a^ | 71.3 ± 2.35_a_ | 149 ± 34.9^a^ | 53.5 ± 1.84_b_ | 100 ± 3.14^a^ | 52.9 ± 1.83_b_ | 106 ± 7.22^a^ | 57.1 ± 3.3_ab_ |  |
| **16:1n-7** | 1.83 ± 1.09^ab^ | 1.08 ± 0.68^ab^ | 12 ± 2.95^a^ | 4.25 ± 0.21^a^ | 3.4 ± 2.31^ab^ | 1.71 ± 1.15^ab^ | 0.27 ± 0.03^b^ | 0.15 ± 0.01^b^ |  |
| **18:1n-9** | 9.59 ± 2.24^a^ | 4.9 ± 0.42_a_ | 14.5 ± 5.87^a^ | 4.67 ± 1.19_ab_ | 5.25 ± 0.12^a^ | 2.76 ± 0.06_b_ | 7.38 ± 0.06^a^ | 3.99 ± 0.01_ab_ |  |
| **20:1n-11** | 7.25 ± 3.62^b^ | 2.99 ± 1.24_b_ | 19.7 ± 1.75^a^ | 8.36 ± 2.34_ab_ | 22.5 ± 1.6^a^ | 11.8 ± 0.91_a_ | 17.6 ± 2.55^ab^ | 9.55 ± 1.48_ab_ |  |
| **∑MUFA** | 25 ± 7.91^a^ | 11.9 ± 1.66_b_ | 57.2 ± 10.2^a^ | 21.1 ± 0.56_a_ | 38.5 ± 2.99^a^ | 20.2 ± 1.22_a_ | 33.9 ± 1.37^a^ | 18.4 ± 0.94_a_ |  |
| **18:3n-6** | 7.4 ± 1.77^a^ | 3.92 ± 0.79_a_ | 17.8 ± 7.01^a^ | 5.76 ± 1.38_a_ | 12.7 ± 0.56^a^ | 6.69 ± 0.43_a_ | 7.99 ± 0.72^a^ | 4.33 ± 0.44_a_ |  |
| **20:4n-6** | 3.71 ± 2.88^b^ | 1.27 ± 0.98_b_ | 12.2 ± 1.73^a^ | 4.59 ± 0.3_a_ | 8.83 ± 0.29^ab^ | 4.64 ± 0.13_a_ | 8.24 ± 0.68^ab^ | 4.46 ± 0.41_ab_ |  |
| **20:5n-3** | 5.89 ± 0.98^b^ | 3.17 ± 0.42_a_ | 11.4 ± 0.72^a^ | 4.79 ± 1.23_a_ | 10.4 ± 0.69^a^ | 5.47 ± 0.36_a_ | 9.56 ± 1.74^ab^ | 5.19 ± 1_a_ |  |
| **22:6n-3** | 3.85 ± 1.25^a^ | 1.89 ± 0.33_b_ | 8.51 ± 0.97^a^ | 3.25 ± 0.31_a_ | 7.84 ± 0.69^a^ | 4.11 ± 0.31_a_ | 6.23 ± 0.56^a^ | 3.37 ± 0.34_a_ |  |
| **∑PUFA** | 33.4 ± 8.92^a^ | 16.8 ± 2.01_b_ | 68 ± 10.7^a^ | 25.4 ± 1.28_a_ | 51.3 ± 1.76^a^ | 27 ± 0.8_a_ | 45.4 ± 3.88^a^ | 24.6 ± 2.36_a_ |  |
| **TOTAL** | 194 ± 38.8^a^ | 100 ± 0_a_ | 275 ± 55.8^a^ | 100 ± 0_a_ | 190 ± 3.98^a^ | 100 ± 0_a_ | 185 ± 1.98^a^ | 100 ± 0_a_ |  |
| **∑n-3 PUFA** | 14.9 ± 2.63^b^ | 7.82 ± 0.49_a_ | 24.8 ± 0.52^a^ | 9.95 ± 1.83_a_ | 21.5 ± 1.49^ab^ | 11.3 ± 0.7_a_ | 19.9 ± 1.9^ab^ | 10.8 ± 1.14_a_ |  |
| **∑n-6 PUFA** | 14.1 ± 2.21^b^ | 7.47 ± 0.46_a_ | 23.4 ± 0.14^a^ | 9.47 ± 1.87_a_ | 21.4 ± 1.44^ab^ | 11.2 ± 0.68_a_ | 19.3 ± 2.29^ab^ | 10.5 ± 1.35_a_ |  |
| **∑n-3 LC PUFA** | 17.6 ± 5.73^a^ | 8.65 ± 1.55_b_ | 41.3 ± 10.1^a^ | 14.7 ± 0.69_a_ | 28.4 ± 0.04^a^ | 14.9 ± 0.3_a_ | 24.3 ± 1.75^a^ | 13.2 ± 1.08_ab_ |  |
| **∑n-6 LC PUFA** | 7.45 ± 3.01^a^ | 3.39 ± 0.71_b_ | 17.6 ± 1.6^a^ | 6.83 ± 0.81_a_ | 13.4 ± 0.31^a^ | 7.07 ± 0.21_a_ | 12.8 ± 1.15^a^ | 6.92 ± 0.7_a_ |  |
| **n-3:n-6** | 1.02 ± 0.19^a^ | 1.02 ± 0.19_a_ | 0.7 ± 0.16^ab^ | 0.7 ± 0.16_ab_ | 0.82 ± 0.02^ab^ | 0.82 ± 0.02_a_ | 1.02 ± 0.01^b^ | 1.02 ± 0.02_a_ |  |
| **LC n-3:LC n-6** | 2.54 ± 0.48^a^ | 2.54 ± 0.48_a_ | 1.35 ± 0.12^ab^ | 1.35 ± 0.12_a_ | 1.5 ± 0.04^a^ | 1.5 ± 0.04_a_ | 1.89 ± 0.04^b^ | 1.89 ± 0.04_a_ |  |
| **EPA:DHA** | 1.53 ± 0.26^ab^ | 1.53 ± 0.26_ab_ | 1.41 ± 0.25^b^ | 1.41 ± 0.25_b_ | 1.5 ± 0.15^b^ | 1.5 ± 0.15_b_ | 1.54 ± 0.48^a^ | 1.54 ± 0.48_a_ |  |
| **EPA:ARA** | 1.56 ± 1.24^a^ | 1.56 ± 1.24_a_ | 1.01 ± 0.2^ab^ | 1.01 ± 0.2_ab_ | 1.14 ± 0.12^ab^ | 1.14 ± 0.12_ab_ | 0.86 ± 0.03^b^ | 0.86 ± 0.03_b_ |  |

Values are presented as means ± SEM. Values in the same row that do not share a superscript are significantly different (*P*<0.05). Values in the same row that do not share a subscript are significantly different (*P*<0.05).
